# Synthesis, Anticancer Activity, and In Silico Studies of 5-(3-Bromophenyl)-*N*-aryl-4*H*-1,2,4-triazol-3-amine Analogs

**DOI:** 10.3390/molecules28196936

**Published:** 2023-10-05

**Authors:** Mohamed Jawed Ahsan, Krishna Gautam, Amena Ali, Abuzer Ali, Abdulmalik Saleh Alfawaz Altamimi, Salahuddin, Manal A. Alossaimi, S. V. V. N. S. M. Lakshmi, Md. Faiyaz Ahsan

**Affiliations:** 1 Department of Pharmaceutical Chemistry, Maharishi Arvind College of Pharmacy, Ambabari Circle, Jaipur 302039, Rajasthan, India; jawedpharma@gmail.com (M.J.A.); gautam.jayesh20@gmail.com (K.G.); 2 Department of Pharmaceutical Chemistry, College of Pharmacy, Taif University, Taif 21944, Saudi Arabia; amrathore@tu.edu.sa; 3 Department of Pharmacognosy, College of Pharmacy, Taif University, Taif 21944, Saudi Arabia; abuali@tu.edu.sa; 4 Department of Pharmaceutical Chemistry, College of Pharmacy, Prince Sattam Bin Abdulaziz University, Al-Kharj 11942, Saudi Arabia; m.alossaimi@psau.edu.sa; 5 Department of Pharmaceutical Chemistry, Noida Institute of Engineering and Technology (Pharmacy Institute), Knowledge Park-2, Greater Noida 201306, Uttar Pradesh, India; sallu_05@yahoo.co.in; 6 Department of Pharmacognosy, Vishnu Institute of Pharmaceutical Education & Research, Narsapur 502313, Medak Dist., Telangana, India; lakshmi.svvnsm@viper.ac.in; 7 Department of Chemistry, Bihar National College, Patna 800004, Bihar, India; faiyaz.ahsan123@gmail.com

**Keywords:** anticancer, ADME studies, cell lines, molecular docking, triazole, tubulin

## Abstract

In the current study, we described the synthesis of ten new 5-(3-Bromophenyl)-*N*-aryl-4*H*-1,2,4-triazol-3-amine analogs (**4a–j**), as well as their characterization, anticancer activity, molecular docking studies, ADME, and toxicity prediction. The title compounds (**4a–j**) were prepared in three steps, starting from substituted anilines in a satisfactory yield, followed by their characterization via spectroscopic techniques. The National Cancer Institute (NCI US) protocol was followed to test the compounds’ (**4a–j**) anticancer activity against nine panels of 58 cancer cell lines at a concentration of 10^−5^ M, and growth percent (GP) as well as percent growth inhibition (PGI) were calculated. Some of the compounds demonstrated significant anticancer activity against a few cancer cell lines. The CNS cancer cell line SNB-75, which showed a PGI of 41.25 percent, was discovered to be the most sensitive cancer cell line to the tested compound **4e**. The mean GP of compound **4i** was found to be the most promising among the series of compounds. The five cancer cell lines that were found to be the most susceptible to compound **4i** were SNB-75, UO-31, CCRF-CEM, EKVX, and OVCAR-5; these five cell lines showed PGIs of 38.94, 30.14, 26.92, 26.61, and 23.12 percent, respectively, at 10^−5^ M. The inhibition of tubulin is one of the primary molecular targets of many anticancer agents; hence, the compounds (**4a–j**) were further subjected to molecular docking studies looking at the tubulin–combretastatin A-4 binding site (PDB ID: 5LYJ) of tubulin. The binding affinities were found to be efficient, ranging from −6.502 to −8.341 kcal/mol, with two major electrostatic interactions observed: H-bond and halogen bond. Ligand **4i** had a binding affinity of −8.149 kcal/mol with the tubulin–combretastatin A-4 binding site and displayed a H-bond interaction with the residue Asn258. The ADME and toxicity prediction studies for each compound were carried out using SwissADME and ProTox-II software. None of the compounds’ ADME predictions showed that they violated Lipinski’s rule of five. All of the compounds were also predicted to have LD_50_ values between 440 and 500 mg/kg, putting them all in class IV toxicity, according to the toxicity prediction. The current discovery could potentially open up the opportunity for further developments in cancer.

## 1. Introduction

**Figure 1:**
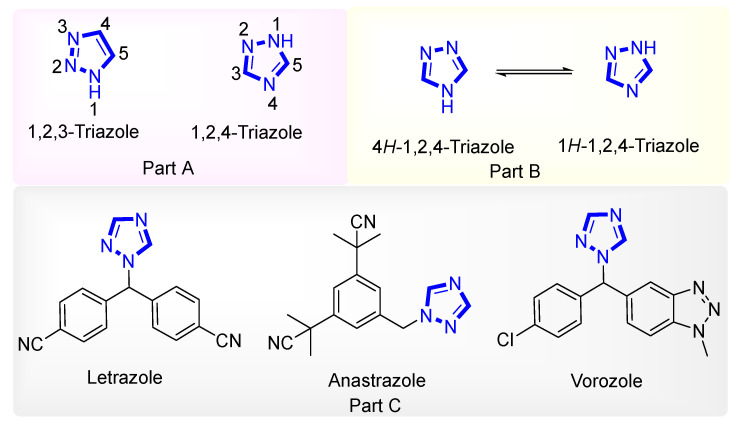
(**A**) Isomeric form of triazole, 1,2,3-triazole, and 1,2,4-triazole; (**B**) 4*H* and 1*H*-Tautomers of 1,2,4-triazole; (**C**) 1,2,4-triazole-containing anticancer agents.

Triazole, with the chemical formula C_2_H_3_N_3_, is one of the main heterocyclic compounds that has three nitrogen atoms in a five-membered ring. There are two potential isomers of triazole, 1,2,3 and 1,2,4-triazoles, depending on where the nitrogen atom is located within the five-membered ring (Figure 1A). It is possible to classify the 1,2,4-triazole ring as an ester, amide, carboxylic acid, or other heterocycle isostere. There may be equilibrium between the 1*H*-form and 4*H*-form for the 1,2,4-triazole ring (Figure 1B) [1,2]. The 1,2,4-triazole-containing ring system is a common pharmacophore incorporated into a wide range of therapeutically interesting active molecules [3]. A triazole heterocyclic ring is an effective and versatile moiety and is among the most studied chemotherapeutic prospects. The presence of numerous commercially available anticancer medications, including anastrozole, vorozole, and letrozole, which contain this scaffold as part of their structural makeup, supports the assertion that compounds possessing 1,2,4-triazoles stand out as the most promising anticancer agents among these two triazole structural isomers (Figure 1C) [3,4,5]. There have been numerous recent studies on 1,2,4-triazole-containing compounds acting as biologically active agents against various diseases, particularly as anticancer agents with apoptosis-inducing abilities [4]. Heterocyclic compounds have long been a fascinating topic due to the ever-increasing need for therapeutic chemicals. The 1*H*-1,2,4-triazole is a useful substitute in bioactive compounds with a variety of pharmacological actions due to its modest dipole character, stiffness, and stability in vivo [6]. Anticancer, antiprotozoal, antibacterial, antiproliferative, β-lactamase-inhibitory, anti-inflammatory, agrochemical, and material studies could all benefit from this moiety. The chemistry of 1,2,4-triazoles has received a lot of attention due to their synthetic utility and diverse range of biological activity. Numerous studies have demonstrated the potent biological properties of 1,2,4-triazoles, including their ability to be analgesic, antibacterial, antimicrobial, antifungal, anticancer, antitubercular, antinociceptive, antioxidant, anticonvulsant, antimycobacterial, antiviral, and anti-inflammatory, as well as show antimycotic activity [7,8,9,10,11,12,13,14,15,16,17,18,19].

**Figure 2:**
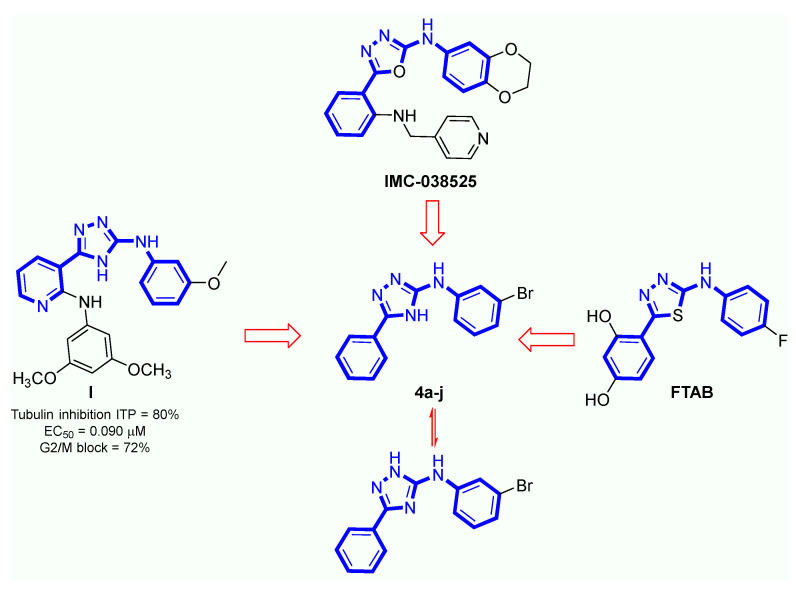
Design of new triazoles based on reported anticancer compounds.

Due to 1,2,4-triazole’s significance in biology, numerous techniques have been developed to create this scaffold, which possesses biological activity. Through multistep synthetic approaches, the synthetic methods documented to date give access to a variety of 1,2,4-triazoles. Surveys of the efficient synthetic techniques to create products containing 1,2,4-triazole systems have rapidly increased in recent studies, in response to the growing demand for the convenient and quick synthesis of heterocycles with biological activity [1]. Given the increasing significance of 1,2,4-triazole in emerging sciences, a thorough evaluation of this unique heterocyclic scaffold utilizing 1,2,4-triazole is required [1]. Due to their well-known pharmacologic efficacy, 1,2,4-triazoles have drawn the interest of numerous research teams looking to develop synthetic drugs with high bioavailability [20]. Compound **I** (reported as the most potent anticancer and anti-tubulin compound), IMC-038525 and FTAB (anticancer compounds), and the triazoles that we report on herein contain structural similarity, as shown in Figure 2 [21,22,23]. We further performed molecular docking studies with 3-Bromophenyl substitution at position 5 of the triazole ring and found efficient binding affinity with the tubulin–combretastatin A-4 binding site (PDB ID: 5LYJ), with the docking score ranging from −6.502 to −8.341 kcal/mol.

Cancer is ranked as one of the most aggressive and lethal diseases across the world. In 2020, GLOBOCAN reported nearly 10 million cancer deaths and approximately 19.3 million new cancer cases [24]. Cancer, if not treated correctly, is likely to become widespread in a large proportion of the world’s population. Existing chemotherapeutic drugs such as cisplatin, doxorubicin, chlorambucil, paclitaxel, and thiotepa fail to inhibit the growth of cancerous cells. This is primarily due to the fact that they are not selective against tumor cells, and secondarily because cancer cells develop resistance due to their unique structural and functional characteristics. In addition, the above-mentioned chemotherapeutic drugs have genotoxic and cytotoxic effects that are detrimental to human soft tissues, causing problems in cancer treatment. As a result, there is an urgent need for a new generation of versatile chemotherapeutic drugs. Thus, anticancer drug research strenuously continues to focus on finding new anticancer therapeutics with low toxicity and enhanced efficacy [6].

## 2. Results

### 2.1. Chemistry

**Scheme 1:**
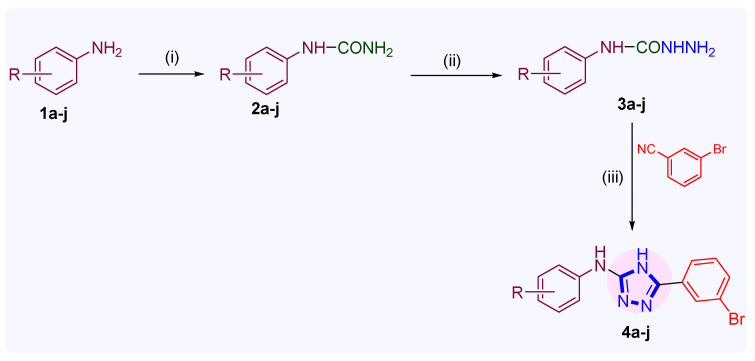
Reagents and conditions: (i). NaCNO, AcOH, 25 °C, 30 min, 82–94% yield; (ii). NH_2_NH_2_.H_2_O, C_2_H_5_OH, 80 °C, 24 h, 69–89%, yield (iii); *n*-Butanol, K_2_CO_3_, 120 °C, 8–10 h, 69–93% yield.

Substituted aniline (**1a–j**) was allowed to react with sodium cyanate via stirring at room temperature to obtain substituted phenyl urea (**2a–j**) in the first step [25]. The substituted phenyl urea (**2a–j**) was refluxed with hydrazine hydrate in ethanol to obtain substituted phenyl semicarbazide (**3a–j**) in the second step [25] (Please refer Supplementary Materials for the general methods of synthesis for the intermediate compounds, **2a–j** and **3a–j**). In the final intermediate step, (**3a–j**) was treated with 3-bromobenzonitrile in *n*-butanol with an addition of potassium carbonate to obtain 5-(3-Bromophenyl)-*N*-aryl-4*H*-1,2,4-triazol-3-amine (**4a–j**) [26,27]. The reaction was monitored via thin-layer chromatography (TLC) using eluent benzene/acetone (9:1). The synthetic protocols, reaction conditions, reagents, and yields in the individual steps are summarized in Scheme 1. The physical constants of the final compounds (**4a–j**) are summarized in Table 1. All of the prepared compounds were prepared in satisfactory yields (65 to 93%) and magnificently characterized via analytical techniques, including nuclear magnetic resonance (NMR), mass spectroscopy, and elemental analysis. The ^1^H NMR of the prototype compound **4a** demonstrated a multiplet at δ ppm 7.39–7.44, corresponding to the H3 and H5 protons of a 4-fluorophenyl ring, two multiplets at δ ppm 7.70–7.73 and 7.76–7.97, corresponding to the H5 and H4 protons of a 3-Bromophenyl ring, respectively, a multiplet at δ ppm 8.04–8.08 for the two protons of the 4-fluorophenyl ring (H3 and H5), and one proton of 3-Bromophenyl (H6). A singlet peak at δ ppm 8.73 was observed for the ArNH, while a singlet at δ ppm 9.13 was observed for triazole NH. The ^13^C NMR revealed twelve different carbons at δ ppm 157.99, 157.53, 156.29, 134.57, 132.85, 131.86, 131.26, 128.62, 126.66, 122.68, 120.80, and 116.43. The ESI-MS of the compounds revealed (M+1)^+^ and (M+2)^+^ peaks at *m*/*z*, 333.01 and 333.99, corresponding to their molecular formula C_14_H_10_BrFN_4_. Please refer Supplementary Materials for the NMR and mass spectra of the compounds (Figures S1–S15).

### 2.2. Anticancer Activity

The compounds’ anticancer activity was observed in nine panels of 58 NCI cancer cell lines at a concentration of 10^−5^ M [28,29,30,31,32]. The anticancer activity was measured in terms of growth percent (GP) and percent growth inhibition (PGI), which are related as PGI=100−GP. The results of anticancer activity in terms of growth control (growth percent) are given in Table 2. The five cell lines that are most sensitive to each compound are shown in Table 3. The renal cancer cell line UO-31 was found to be the most sensitive against six compounds **4a**, **4b**, **4c**, **4f**, **4h,** and **4j**, with a PGI of 26.68, 31.14, 26.47, 37.17, 36.57, and 33.43 percent, respectively, while UO-31 was found to be the second most sensitive cell line against the compounds **4d**, **4e**, **4g**, and **4i**, with a PGI of 32.2, 28.47, 20.23, and 30.14, respectively. The non-small cell lung cancer cell line EKVX was the second most sensitive cell line against five compounds **4a**, **4b**, **4f**, **4h**, and **4j**, with a PGI of 21.72, 19.83, 17.03, 24.56, and 24.57 percent, respectively. The CNS cancer cell line SNB-75 was found to be the most sensitive against the compounds **4e**, **4g**, and **4h**, with a PGI of 41.25, 30.09, and 38.94 percent, respectively, while the breast cancer cell line MCF-7 was found to be the most sensitive cell line against compound **4d**. The SNB-75 cancer cell line, which showed a PGI of 41.25 percent, was discovered to be the most sensitive cancer cell line to the tested compound **4e**. The mean growth percent (GP = 97.48) of compound **4i** was recorded as the most significant among the series of compounds and the five cell lines SNB-75, UO-31, CCRF-CEM, EKVX, and OVCAR-5 that showed maximum sensitivity with a PGI of 38.94, 30.14, 26.92, 26.61, and 23.12, respectively. Furthermore, the average PGIs of each cancer cell line panels were calculated and the anticancer activity was compared with the reference drug, imatinib. Compound **4i** with the most significant anticancer activity among the series of ten compounds showed better anticancer activity than imatinib against CNS, melanoma, and ovarian cancer cell lines (Table 4). The anticancer data were retrieved form the National Cancer Institute website [28]. Please refer Supplementary Materials for the anticancer screening data of the compounds **4a–j** at 10^−5^ M (Figures S16–S25). Compound **4i** with 2,6-dimethyl substitution demonstrated good anticancer activity, followed by compound **4d** with 4-methoxy substitution and compound **4e** with 2-chloro substitution. The overall anticancer activity followed, with substitutions of 2,6-dimethyl < 4-methoxy < 2-chloro < 3-chloro-4-fluoro < 2,6-dimethyl < 4-fluoro < 4-chloro < 2-methyl < 4-methyl < 2-methoxy.

### 2.3. Molecular Docking Studies

**Figure 3:**
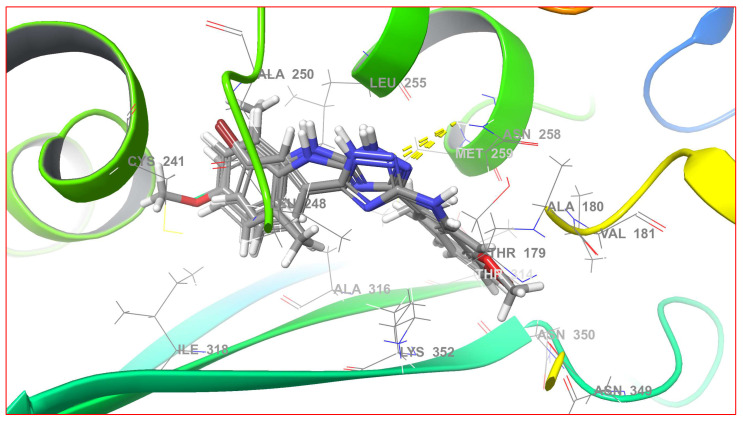
Three-dimensional interaction of ligands **4a**, **4d**, **4f**, **4g**, and **4i** with the tubulin–combretastatin A-4 binding site (PDB ID: 5LYJ).

**Figure 4:**
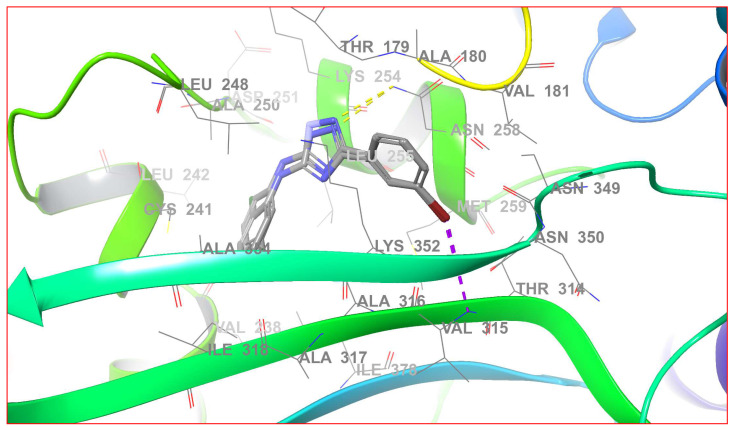
Three-dimensional interaction of ligands **4e** and **4j** with the tubulin–combretastatin A-4 binding site.

**Figure 5:**
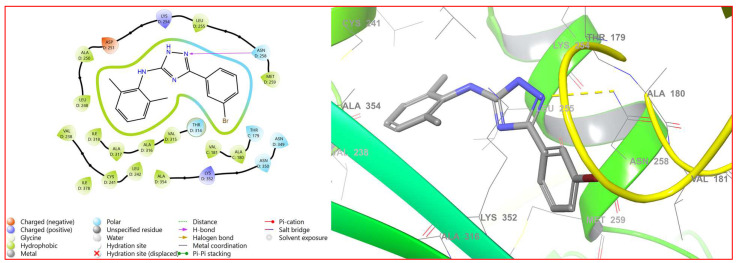
Two- and three-dimensional interaction of ligand **4i** with the tubulin-combretastatin A-4 binding site.

One of the key molecular targets of many anticancer drugs is the inhibition of tubulin. Colchicine, combretastatin A-4, epothilones, nocodazole, vinca alkaloids, and taxanes are a few examples of tubulin inhibitors used in cancer chemotherapy [33]. The molecular docking of the ligands (**4a–j**) was carried out against the tubulin–combretastatin A-4 binding site (PDB ID: 5LYJ), and the results are given in Table 5. The docking scores ranged from −6.502 to −8.341 kcal/mol, with two major electrostatic interactions observed: H-bond and halogen bond. The ligands **4a**, **4d**, **4f**, **4g**, and **4i** demonstrated a similar type of interaction and displayed a H-bond interaction with the residue Asn258 (Figure 3). The ligands **4e** and **4j** displayed a similar type of interaction, a H-bond interaction with the residue Asn258 and a halogen bond interaction with the residue Val315 (Figure 4), while ligand **4b** demonstrated a H-bond interaction with the residue Asn258 and a halogen bond interaction with the residue Cys241 (Figure 5). The molecular docking studies showed that ligand **4i** had a binding affinity of −8.149 kcal/mol and displayed a H-bond interaction with the residue Asn258. The two- and three-dimensional interactions of ligand **4i** with the tubulin–combretastatin A-4 binding site are shown in Figure 5.

### 2.4. ADME and Toxicity Prediction Studies

**Figure 6:**
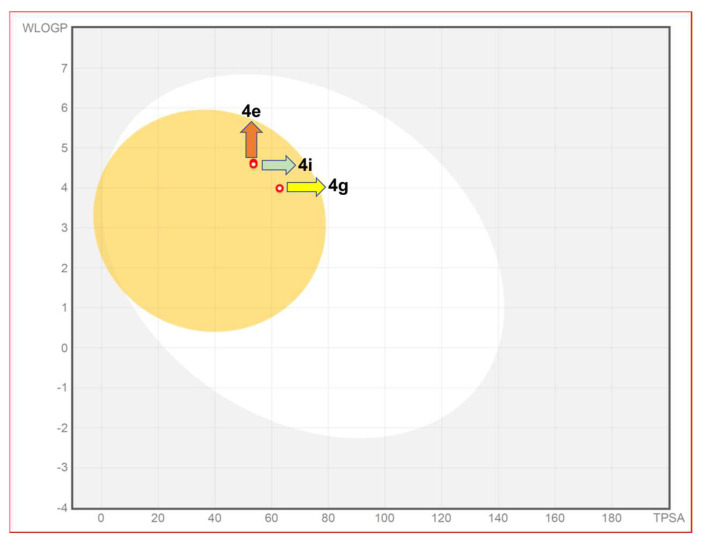
Boiled egg presentation of compounds **4e**, **4g**, and **4i** using SwissADME software.

Absorption, distribution, metabolism, and excretion, also known as ADME, are crucial variables to examine during the drug discovery process in order to reduce the likelihood of pharmacokinetics-related clinical failure [34]. The ADME prediction via SwissADME software [35] showed that all of the compounds followed Lipinski’s rule of five, with their molecular weight ranging from 329.19 to 367.6 (MW < 500), log *p* values ranging from 2.15 to 2.48 (log *p* < 5), number of hydrogen bond acceptors (HBAs) ranging from 2 to 3 (HBA < 10), and number of hydrogen bond donors (HBDs) being two (HBDs < 5) [36]. The percent absorption was calculated from the equation % Abs=109±[0.345×TPSA], and was found to be 87.32 to 90.50 percent [37]. Lipinski’s rule of five is a general rule of thumb for evaluating druglikeness or determining whether a chemical compound with particular pharmacological or biological activity has physicochemical properties that would probably make it an orally active drug in humans. Furthermore, the compounds were found to be blood–brain barrier (BBB)-permeant in SwissADME prediction, as shown vis the boiled egg representation of compounds **4e**, **4g**, and **4i** (Figure 6). The physicochemical space of molecules with the highest probability of entering the gastrointestinal tract is represented by the white region, and the physicochemical space of molecules with the highest probability of entering the brain is represented by the yellow region (yolk) [38]. The compounds **4e**, **4g**, and **4i** were predicted to be highly absorbed from the gastrointestinal tract (GIT) and enter the BBB efficiently. Pharmaceutical companies experience significant financial losses as a result of post-marketing drug failure brought on by ADME and toxicity [39]. Toxicity prediction by ProTox-II predicted the toxicity with LD_50_ values between 440 and 500 mg/kg, putting them all in the class IV toxicity class (300 < LD_50_ ≤ 2000) [40]. The ADME and toxicity prediction of compounds (**4a–j**) are given in Table 6.

## 3. Discussion

5-(3-Bromophenyl)-*N*-aryl-4*H*-1,2,4-triazol-3-amine (**4a–j**) was prepared in three steps starting from substituted anilines (**1a–j**). Substituted phenyl urea (**2a–j**) and *N*-(substituted pheny)hydrazine carboxamide (**3a–j**) were prepared as intermediate compounds using a well-established synthetic procedure, as reported earlier [25]. The title compounds (**4a–j**) were prepared with a good yield and were thoroughly characterized using spectroscopic methods of analysis followed by their anticancer evaluation against 58 cancer cell lines in a one-dose assay at 10 µM. The CNS cancer cell line SNB-75 was found to be the most sensitive against compound **4e** (PGI = 41.25). Compound **4i** demonstrated the most significant anticancer activity with a mean GP of 97.48. SNB-75, UO-31, CCRF-CEM, EKVX, and OVCAR-5 were the five most sensitive cell lines to compound **4i**, with PGIs of 38.94, 30.14, 26.92, 26.61, and 23.12, respectively. In comparison to the previously reported compound, the lead compound **4i** had better anticancer effects on cancer cell lines. Because the prepared compounds shared the basic pharmacophore of previously reported anticancer and anti-tubulin compounds, tubulin was chosen as a plausible molecular target, and molecular docking against tubulin was examined to see whether any interactions could be observed. All of the compounds demonstrated efficient binding with the tubulin–combretastatin A-4 binding site (PDB ID: 5LYJ), with docking scores ranging from −6.502 to −8.341 kcal/mol and two major types of electrostatic interactions being observed: H-bond (with Asn258) and halogen bond (with Cys241 and Val315). The ADME studies are vital parameters to study during the drug discovery process in order to reduce the probability of pharmacokinetics-related clinical failure. Post-marketing drug failure brought on by ADME and toxicity results in significant financial losses for pharmaceutical companies. Therefore, when developing novel compounds with therapeutic value, ADME and toxicity studies should not be ignored. All of the compounds were consequently investigated for ADME and toxicity prediction. According to swissADME prediction, all of the compounds were found to be BBB-permeant. The compounds **4e**, **4g**, and **4i** showed promising anticancer activity against the CNS cancer cell line SNB-75, and swissADME prediction studies revealed that these compounds are BBB-permeant. Lipinski’s rule of five is a general guideline for assessing druglikeness or determining whether a chemical compound has physicochemical properties that would probably make it an orally active drug in humans. According to the rule, a compound must meet five requirements in order to function as an oral drug: a molecular weight of ≤500, a log *p* of <5, an HBA of <10, and an HBD of <5. None of the compounds violated Lipinski’s rule of five, and ProTox-II predicted toxicity with LD_50_ values ranging from 440 to 500 mg/kg, putting them all in class IV toxicity. The current discovery could pave the way for the development of improved triazoles through chemical modification, leading to future advances in cancer therapeutics.

## 4. Materials and Methods

### 4.1. General Method for the Synthesis of 5-(3-Bromophenyl)-N-aryl-4H-1,2,4-triazol-3-amine analogs (***4a–j***)

Equimolar amounts of *N*-(substituted phenyl)hydrazine carboxamide (**3a–j**) (1 mmol) and 3-bromobenzonitrile (1 mmol; 182 mg) were dissolved in *n*-butanol (10 mL), and K_2_CO_3_ (1 g) was added and heated at 120 °C with continuous stirring via a magnetic stirrer for 8 h. Excess of solvent was removed, the reaction mixture was poured into the crushed ice, and the product was extracted in ethyl acetate; later, it was separated via filtration flask, obtained as a solid product of 5-(3-Bromophenyl)-*N*-aryl-4*H*-1,2,4-triazol-3-amine analogs (**4a–j**), and re-crystallized from petroleum ether [26,27].

5-(3-Bromophenyl)-*N*-(4-fluorophenyl)-4H-1,2,4-triazol-3-amine (**4a**): ^1^H NMR (300 MHz, DMSO-*d*_6_): δ ppm: 7.39–7.44 (m, 2H, ArH), 7.49 (s, 1H, ArH), 7.70–7.73 (m, 1H, ArH), 7.76–7.97 (m, 1H, ArH), 8.04–8.08 (m, 3H, ArH), 8.73 (s, 1H, ArNH), 9.13 (s, 1H, NH); ^13^C NMR (75 MHz, DMSO-*d*_6_): δ ppm: 157.99, 157.53, 156.29, 134.57, 132.85, 131.86, 131.26, 128.62, 126.66, 122.68, 120.80, 116.43; anal. calc. for C_14_H_10_BrFN_4_: C, 50.47; H, 3.03; N, 16.82 found: C, 50.30; H, 3.01; N, 16.75%. ESI-MS *m*/*z* = 333.01 (M+1)^+^, 333.99 (M+2)^+^.

5-(3-Bromophenyl)-*N*-(4-chlorophenyl)-4H-1,2,4-triazol-3-amine (**4b**): ^1^H NMR (300 MHz, DMSO-*d*_6_): δ ppm: 7.19 (d, *J* = 6.0 Hz, ArH), 7.39–7.43 (m, 2H, ArH), 7.49 (s, 1H, ArH), 7.70–73 (m, 1H, ArH), 7.76–7.97 (m, 1H, ArH), 8.04–8.08 (m, 3H, ArH), 8.73 (s, 1H, ArNH), 9.13 (s, 1H, NH); ^13^C NMR (75 MHz, DMSO-*d*_6_): δ ppm: 157.99, 157.39, 156.40, 134.64, 132.91, 131.93, 131.49, 128.48, 126.66, 122.68, 120.80, 116.43; anal. calc. for C_14_H_10_BrClN_4_: C, 48.10; H, 2.88; N, 16.03 found: C, 48.02; H, 2.86; N, 16.00%. ESI-MS *m*/*z* = 348.94 (M+1)^+^, 350.90 (M+3)^+^.

5-(3-Bromophenyl)-*N*-(*p*-tolyl)-4H-1,2,4-triazol-3-amine (**4c**): ^1^H NMR (300 MHz, DMSO-*d*_6_): δ ppm: 2.32 (s, 3H, CH_3_), 7.36–7.54 (m 1H, ArH), 7.59 (s, 1H, ArH), 7.83 (d, *J* = 12.3 Hz, 2H, ArH), 7.94 (d, *J* = 14.0 Hz, 2H, ArH), 8.01–8.11 (m, 2H, ArH), 8.32 (s, 1H, ArNH), 8.64 (s, 1H, NH); ^13^C NMR (75 MHz, DMSO-*d*_6_): δ ppm: 158.62, 156.63, 135.59, 132.90, 131.79, 131.54, 131.24, 129.89, 126.16, 126.65, 122.27, 120.34, 21.35; anal. calc. for C_15_H_13_BrN_4_: C, 54.73; H, 3.98; N, 17.02 found: C, 54.65; H, 3.95; N, 16.98%. ESI-MS *m*/*z* = 329.10 (M+1)^+^.

5-(3-Bromophenyl)-*N*-(4-methoxyphenyl)-4H-1,2,4-triazol-3-amine (**4d**): ^1^H NMR (300 MHz, DMSO-*d*_6_): δ ppm: 3.81 (s, 3H, ArH), 6.93 (d, *J* = 6.0 Hz, 2H, ArH), 7.77 (d, *J* = 6.3 Hz, 2H, ArH), 7.58 (s, 1H, ArH), 7.39–7.43 (m, 1H, ArH), 7.65–7.68 (m, 1H, ArH), 8.05–8.12 (m, 1H, ArH), 8.33 (s, 1H, ArNH), 8.89 (s, 1H, NH); ^13^C NMR (75 MHz, DMSO-*d*_6_): δ ppm: 157.91, 156.21, 153.93, 132.89, 131.79, 131.47, 131.19, 128.18, 126.57, 121.72, 115.16, 55.82; anal. calc. for C_15_H_13_BrN_4_O: C, 52.19; H, 3.80; N, 16.23 found: C, 52.09; H, 3.75; N, 16.18%. ESI-MS *m*/*z* = 345.24 (M+1)^+^.

5-(3-Bromophenyl)-*N*-(2-chlorophenyl)-4H-1,2,4-triazol-3-amine (**4e**): ^1^H NMR (300 MHz, DMSO-*d*_6_): δ ppm: 7.39–7.44 (m, 2H, ArH), 7.53 (s, 1H, ArH), 7.70–7.73 (m, 1H, ArH), 7.73–7.80 (m, 2H, ArH), 7.95–8.12 (m, 2H, ArH), 8.32 (s, 1H, ArNH), 8.55 (s, 1H, NH); ^13^C NMR (75 MHz, DMSO-*d*_6_): δ ppm: 157.62, 156.63, 136.79, 132.91, 131.71, 131.44, 130.27, 129.89, 128.33, 127.76, 126.51, 125.47, 122.94, 122.25; anal. calc. for C_14_H_10_BrClN_4_: C, 48.10; H, 2.88; N, 16.03 found: C, 48.03; H, 2.86; N, 15.99%. ESI-MS *m*/*z* = 348.94 (M+1)^+^, 350.90 (M+3)^+^.

5-(3-Bromophenyl)-*N*-(*o*-tolyl)-4H-1,2,4-triazol-3-amine (**4f**): ^1^H NMR (300 MHz, DMSO-*d*_6_): δ ppm: 2.12 (s, 3H, CH_3_), 7.35–7.44 (m, 2H, ArH), 7.48 (s, 1H, ArH), 7.70–7.73 (m, 1H, ArH), 7.84–7.94 (m, 2H, ArH), 8.04–8.07 (m, 2H, ArH), 8.30 (s, 1H, ArNH), 8.80 (s, 1H, NH); ^13^C NMR (75 MHz, DMSO-*d*_6_): δ ppm: 157.68, 156.69, 142.19, 132.91, 131.77, 131.49, 131.37, 129.09, 128.13, 126.96, 126.11, 123.97, 123.34, 122.29, 18.42; anal. calc. for C_15_H_13_BrN_4_: C, 54.73; H, 3.98; N, 17.02 found: C, 54.69; H, 3.96; N, 16.97%. ESI-MS *m*/*z* = 329.06 (M+1)^+^.

5-(3-Bromophenyl)-*N*-(2-methoxylphenyl)-4H-1,2,4-triazol-3-amine (**4g**): ^1^H NMR (300 MHz, DMSO-*d*_6_): δ ppm: 3.86 (s, 3H, OCH_3_), 6.97–7.21 (m, 4H, ArH), 7.39–7.42 (m, 1H, ArH), 7.58 (s, 1H, ArH), 7.72–7.75 (1H, m, ArH), 8.02–8.96 (m, 1H, ArH), 8.39 (s, 1H, ArNH), 9.989 (s, 1H, ArH); ^13^C NMR (75 MHz, DMSO-*d*_6_): δ ppm: 157.72, 156.81, 147.52, 132.99, 132.27, 131.97, 131.57, 128.11, 126.89, 122.99, 122.14, 121.19, 113.89, 112.82, 56.62; anal. calc. for C_15_H_13_BrN_4_O: C, 52.19; H, 3.80; N, 16.23 found: C, 52.10; H, 3.76; N, 16.19%. ESI-MS *m*/*z* = 345.04 (M+1)^+^.

5-(3-Bromophenyl)-*N*-(2,4-dimethylphenyl)-4H-1,2,4-triazol-3-amine (**4h**): ^1^H NMR (300 MHz, DMSO-*d*_6_): δ ppm: 2.14 (s, 3H, CH_3_), 2.24 (s, 3H, CH_3_), 6.84–7.06 (m, 1H, ArH), 7.39–7.56 (m, 2H, ArH), 7.72 (d, *J* = 8.7 Hz, 1H, ArH), 7.87 (d, *J* = 7.8 Hz, 1H, ArH), 8.05 (s, 1H, ArH), 8.15 (s, 1H, ArH), 8.32 (s, 1H, ArNH), 9.65 (s, 1H, NH); ^13^C NMR (75 MHz, DMSO-*d*_6_): δ ppm: 157.99, 156.91, 139.19, 137.87, 132.97, 131.93, 131.59, 131.19, 128.91, 128.14, 126.89, 126.59, 116.82, 24.18, 18.42; anal. calc. for C_16_H_15_BrN_4_: C, 55.99; H, 4.41; N, 16.32 found: C, 55.89; H, 4.39; N, 16.28%. ESI-MS *m*/*z* = 343.05 (M+1)^+^.

5-(3-Bromophenyl)-*N*-(2,6-dimethylphenyl)-4H-1,2,4-triazol-3-amine (**4i**): ^1^H NMR (300 MHz, DMSO-*d*_6_): δ ppm: 2.12 (s, 6H, ArH), 7.04–7.14 (m, 3H, ArH), 7.38–7.41 (m. 1H, ArH), 7.59 (s, 1H, ArH), 7.71–7.74 (m, 1H, ArH), 8.04–8.06 (m, 1H, ArH), 8.37 (s, 1H, ArNH), 8.79 (s, 1H, NH); ^13^C NMR (75 MHz, DMSO-*d*_6_): δ ppm: 157.99, 156.53, 137.88, 136.23, 132.91, 131.86, 131.49, 128.57, 128.33, 126.60, 122.83, 121.48, 18.57; anal. calc. for C_16_H_15_BrN_4_: C, 55.99; H, 4.41; N, 16.32 found: C, 55.90; H, 4.40; N, 16.29%. ESI-MS *m*/*z* = 343.06 (M+1)^+^.

5-(3-Bromophenyl)-*N*-(3-chloro-4-fluorophenyl)-4H-1,2,4-triazol-3-amine (**4j**): ^1^H NMR (300 MHz, DMSO-*d*_6_): δ ppm: 7.18–7.23 (m, 2H, ArH), 7.37, (s, 1H, ArH), 7.39–7.41 (m, 1H, ArH), 7.53 (s, 1H, ArH), 7.69–7.71 (m, 1H, ArH), 8.12–8.14 (m, 1H, ArH), 8.36 (s, 1H, NH), 8.89 (s, 1H, ArOH); ^13^C NMR (75 MHz, DMSO-*d*_6_): δ ppm: 157.99, 156.33, 148.88, 139.62, 132.62, 131.42, 128.70, 126.60, 122.83, 121.48, 118.92, 118.32, 114.55; anal. calc. for C_14_H_9_BrClFN_4_: C, 45.74; H, 2.47; N, 15.24 found: C, 45.70; H, 2.45; N, 15.19%. ESI-MS *m*/*z* = 367.60 (M+1)^+^, 369.60 (M+3)^+^.

### 4.2. Anticancer Activity

All of the title compounds were evaluated against 56 cancer cell lines derived from nine panels at 10 µM, as per the reported NCI US protocol [28,29,30,31,32]. A detailed methodology was described in our previous work [41].

### 4.3. Molecular Docking

The X-ray crystallographic structure of the tubulin–combretastatin A-4 complex, with a resolution of 2.40 Å and an R-value of 0.192 (observed), was obtained from the protein data bank (PDB ID: 5LYJ) [42]. The D-chain of the combretastatin A-4 binding site was selected for docking studies. The molecular docking was performed as explained in previous work [43,44].

### 4.4. ADME and Toxicity Prediction

The prediction of ADME was accomplished using SwissADME software available online [35]. The toxicity prediction in terms of LD_50_ and toxicity class was made using ProTox-II [40].

## 5. Conclusions

All of the compounds were successfully synthesized in satisfactory yields, characterized via a spectroscopic method of analysis, anticancer evaluation at 10 µM, and in silico studies. Some of the compounds demonstrated promising anticancer activity against a few cancer cell lines. The CNS cancer cell line SNB-75 was found to be the most sensitive against compound **4e** (PGI = 41.25). With a mean GP of 97.48, the lead compound **4i** showed the strongest anticancer activity. The five cell lines with the highest sensitivity to compound **4i** were SNB-75, UO-31, CCRF-CEM, EKVX, and OVCAR-5, with PGIs of 38.94, 30.14, 26.92, 26.61, and 23.12, respectively. Compound **4i** with 2,6-dimethyl substitution demonstrated good anticancer activity. The molecular docking of the compounds (**4a–j**) against the tubulin–combretastatin A-4 binding site (PDB ID: 5LYJ) was studied and the H-bond and halogen bond were the two main electrostatic interactions seen; the binding affinities were found ranging between −6.502 and −8.341 kcal/mol. The ADME prediction showed that all of the compounds followed Lipinski’s rule of five and were BBB-permeant. ProTox-II’s toxicity prediction indicated that the toxicity had LD_50_ values between 440 and 500 mg/kg, classifying them all as having class IV toxicity (300 > LD_50_ > 2000). The current discovery may pave the way to the chemical synthesis of improved triazoles, resulting in future advancements in cancer therapeutics.

## Figures and Tables

**Table 1:** The physical constants and NSC code of 5-(3-Bromophenyl)-*N*-aryl-4*H*-1,2,4-triazol-3-amine analogs (**4a–j**).

		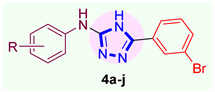			
**S. No.**	**Compound**	**R**	**% Yield**	**Mp (°C)**	R_f_ *
1	**4a**	4-Fluoro	69	138–140	0.58
2	**4b**	4-Chloro	93	140–142	0.66
3	**4c**	4-Methyl	82	136–138	0.73
4	**4d**	4-Methoxy	66	146–148	0.67
5	**4e**	2-Chloro	70	152–154	0.69
6	**4f**	2-Methyl	85	144–146	0.75
7	**4g**	2-Methoxy	65	148–150	0.62
8	**4h**	2,4-Dimethyl	71	130–132	0.70
9	**4i**	2,6-Dimethyl	89	126–128	0.68
10	**4j**	3-Chloro-4-fluoro	70	128–130	0.59

* Mobile phase, benzene/acetone (9:1).

**Table 2:** Anticancer activity of triazoles at 10^−5^ M.

Panel	Cell Lines	Growth Percentage (GP) of Cell Lines at 10^−5^ M									
		4a	4b	4c	4d	4e	4f	4g	4h	4i	4j
*Leukemia*	CCRF-CEM	108.27	119.99	108.68	102.09	94.53	100.88	89.67	101.76	73.08	91.67
	HL-60(TB)	113.71	114.64	111.07	112.68	110.98	110.97	112	113.23	97.14	114.35
	K-562	112.26	109.82	107.94	103.69	105.66	102.97	109.72	103.2	102.19	103.41
	MOLT-4	107.03	108.06	111.15	110.86	113.17	101.46	110.07	102.63	97.59	97.05
	RPMI-8226	105.59	105.54	100.45	98.15	98.41	101.25	95.44	99.56	95.61	97.9
	SR	102.38	101.37	98.98	101.01	100.58	99.66	101.9	96.81	95.05	90.22
*Non-small cell lung cancer*	A549/ATCC	103.16	103.69	105.55	100.84	96.1	100.93	87.98	104.32	93.63	106.05
	EKVX	78.28	80.17	82.04	69.71	79.19	82.97	88.78	75.44	73.39	75.43
	HOP-62	101.06	95.97	101.27	92.36	100.44	95.2	105.98	88.36	84.31	89.96
	NCI-H226	93.31	97.92	100.07	95.53	99.37	104.72	103.45	94.95	92.33	98.69
	NCI-H23	88.7	92.08	93.16	90.01	93.77	92.05	100.71	85.13	90.72	86.94
	NCI-H322M	94.81	98.59	104.27	102.66	103.52	104.54	104.29	90.57	99.97	99.48
	NCI-H460	107.43	109.66	106.66	102.46	105.58	111.91	106.39	108.59	100.48	106.13
	NCI-H522	97.48	94	95.75	96.15	94.51	102.9	99.38	99.53	92.22	96.13
*Colon cancer*	COLO 205	110.19	112.57	110.57	107.34	110.79	113.84	112.68	115.88	107.53	115.25
	HCC-2998	115.61	110.62	111.63	119.85	103.1	108.79	120.05	109.12	117.6	107.2
	HCT-116	97.94	111.89	113.68	107.29	109.62	110.01	109.61	112.05	117.94	108.97
	HCT-15	104.59	102.35	107.09	101.37	102.9	98.06	107.29	101.49	105.41	98.35
	HT29	105.5	112.5	108.16	106.95	108.98	110.4	109.4	105.72	109.17	108.45
	KM12	105.22	112.61	105.97	102.43	100.98	101.9	104.37	99.84	106.75	98.24
	SW-620	105.46	104.34	112.08	96.23	103.98	103.77	102.34	108.99	92.19	107.34
*CNS cancer*	SF-268	106.54	100.84	97.75	95.5	92.22	106.7	99.08	106.04	95.37	108.16
	SF-295	95.3	95.66	95.57	96.26	96.71	96.01	101.31	91.54	97.66	90.8
	SF-539	100.77	99.9	103.01	103.1	104.07	102.34	116.34	95.16	101.64	97.27
	SNB-19	101.28	104.36	105.52	102.63	103.71	97.3	104.02	94.59	96.04	93.01
	SNB-75	85.12	94.98	93.57	74.12	58.75	105.1	69.91	97.86	61.06	87.08
	U251	100.51	98.72	101.28	103.97	95.73	101.07	100.21	103.11	99.09	103.42
*Melanoma*	LOX IMVI	102.66	101.4	101.31	100.89	102.44	99.16	104.8	92.92	95.05	94.16
	MALME-3M	98.54	100.02	96.32	94.93	92.71	91.35	100.59	86.17	90.31	84.86
	M14	105.99	108.76	104.25	112.38	123.95	105.19	116.15	105.2	98.01	111.26
	MDA-MB-435	106.28	107.8	105.37	104.17	104.41	110.41	105.09	108.55	102.27	108.5
	SK-MEL-2	113.88	107.21	103.44	101.76	103.4	107.78	108.78	108.2	97.14	103.77
	SK-MEL-28	113.3	112.88	118.51	108.94	120.72	115.05	120.5	112.62	112.3	117.18
	SK-MEL-5	99.6	98.99	99.5	99.12	99.9	101.68	101.97	100.2	99.65	100.08
	UACC-257	109.84	114.31	110.07	103.12	92.92	103.83	91.52	103.5	91.31	109.37
	UACC-62	86.63	86.88	89.2	86.37	90.47	91.67	91.39	86.81	81.5	82.62
*Ovarian cancer*	IGROV1	97.79	93.46	101.73	96.31	104.69	93.62	108.53	98.08	95.53	95.01
	OVCAR-3	100.18	100.05	102.02	97.2	101.46	108.69	106.81	105.94	101.9	114.63
	OVCAR-4	111.22	110.06	107.14	106.18	101.66	114.27	104.94	117.35	102.73	114.79
	OVCAR-5	97.87	94.97	107.49	104.3	114.17	103.71	110.58	100.92	76.88	95.79
	OVCAR-8	106.58	103.24	106.89	100.78	96.44	103.67	99.3	104.87	97.38	107.02
	NCI/ADR-RES	109.03	107.66	105.99	105.54	109.73	105.49	111.9	103.85	100.18	103.94
	SK-OV-3	119.91	111.75	110.49	102.74	117.29	103.01	117.44	86.52	99.76	104.47
*Renal cancer*	786-0	113.02	103.97	103.28	108.45	102.12	106.4	112	100.76	101.23	98.23
	A498	135.1	134.22	128.18	128.7	111.35	126.93	121.95	133.39	116.19	120.55
	ACHN	114.05	106.61	110.86	102.05	101.53	111.23	110.31	108.67	97.5	106.13
	CAKI-1	85.93	82.74	87.09	82.86	88.17	93.49	90.08	94.18	82.44	97.73
	RXF 393	116.32	117.62	111.11	130.25	117.52	108.82	122.22	108.85	119.36	115.41
	SN12C	102.39	106.19	107.4	100.67	98.2	105.96	103.38	99.06	98.18	97.5
	TK-10	120.17	137.39	130.44	157.41	113.5	127.11	108.98	126.78	127.43	144.83
	UO-31	73.32	68.86	73.53	67.8	71.53	62.83	79.77	63.43	69.86	66.57
*Prostate cancer*	DU-195	104.93	109.62	101.84	99.17	95.28	114.08	102.88	117.08	101.74	107.28
*Breast cancer*	MCF-7	87.72	80.84	81.89	64.81	88.98	88.37	92.82	88.4	91.88	75.48
	MDA-MB-231/ATCC	90.56	89.44	94.04	88.27	95.95	91.79	97.91	88.64	82.84	88.16
	HS 578T	99.43	99.85	108.56	100.87	95	105.79	97.66	113.29	99.85	105.98
	BT-549	95.99	107.15	119.87	101.94	108.77	136.12	122.63	114.36	116.88	120.26
	T-47D	110.59	111.95	106.74	92.6	99.07	115.23	104.25	100.03	103.99	104.12
	MDA-MB-468	105.73	108.68	109.79	104.93	105.76	105.16	109.06	101.61	107.16	96.56
Mean		103.14	103.75	104.09	100.88	100.97	103.89	104.29	101.48	97.48	101.19
Delta		29.82	34.89	30.56	36.07	42.22	41.06	34.38	38.05	36.42	34.62
Range		61.78	68.53	56.91	92.60	65.20	73.29	52.72	69.96	66.37	78.26

**Table 3:** The anticancer activity of the six most sensitive cell lines at 10^−5^ M.

Compound	Anticancer Activity in One Dose (10^−5^ M Concentration)		
	The Most Sensitive Cell Lines	Growth Percent (GP)	Percent Growth Inhibition (PGI)
**4a**	UO-31 (renal cancer)	73.32	26.68
	EKVX (non-small cell lung cancer)	78.28	21.72
	SNB-75 (CNS cancer)	85.12	14.88
	CAKI-1 (renal cancer)	85.93	14.07
	UACC-62 (melanoma)	86.63	13.37
**4b**	UO-31 (renal cancer)	68.86	31.14
	EKVX (non-small cell lung cancer)	80.17	19.83
	MCF-7 (breast cancer)	80.84	19.16
	CAKI-1 (renal cancer)	82.74	17.26
	UACC-62 (melanoma)	86.88	13.12
**4c**	UO-31 (renal cancer)	73.53	26.47
	MCF-7 (breast cancer)	81.89	18.11
	EKVX (non-small cell lung cancer)	82.04	17.96
	CAKI-1 (renal cancer)	87.09	12.91
	UACC-62 (melanoma)	89.2	10.8
**4d**	MCF-7 (breast cancer)	64.81	35.19
	UO-31 (renal cancer)	67.8	32.2
	EKVX (non-small cell lung cancer)	69.71	30.29
	SNB-75 (CNS cancer)	74.12	25.88
	CAKI-1 (renal cancer)	82.86	17.14
**4e**	SNB-75 (CNS cancer)	58.75	41.25
	UO-31 (renal cancer)	71.53	28.47
	EKVX (non-small cell lung cancer)	79.19	20.81
	CAKI-1 (renal cancer)	88.17	11.83
	MCF-7 (breast cancer)	88.98	11.02
**4f**	UO-31 (renal cancer)	62.83	37.17
	EKVX (non-small cell lung cancer)	82.97	17.03
	MCF-7 (breast cancer)	88.37	11.63
	MALME-3M (melanoma)	91.35	8.65
	UACC-62 (melanoma)	91.67	8.33
**4g**	SNB-75 (CNS cancer)	69.91	30.09
	UO-31 (renal cancer)	79.77	20.23
	A549/ATCC (non-small cell lung cancer)	87.98	12.02
	EKVX (non-small cell lung cancer)	88.78	11.22
	CCRF-CEM (leukemia)	89.67	10.33
**4h**	UO-31 (renal cancer)	63.43	36.57
	EKVX (non-small cell lung cancer)	75.44	24.56
	NCI-H23 (non-small cell lung cancer)	85.13	14.87
	MALME-3M (melanoma)	86.17	13.83
	SK-OV-3 (ovarian cancer)	86.52	13.48
**4i**	SNB-75 (CNS cancer)	61.06	38.94
	UO-31 (renal cancer)	69.86	30.14
	CCRF-CEM (leukemia)	73.08	26.92
	EKVX (non-small cell lung cancer)	73.39	26.61
	OVCAR-5 (ovarian cancer)	76.88	23.12
**4j**	UO-31 (renal cancer)	66.57	33.43
	EKVX (non-small cell lung cancer)	75.43	24.57
	MCF-7 (breast cancer)	75.48	24.52
	UACC-62 (melanoma)	82.62	17.38
	MALME-3M (melanoma)	84.86	15.14

**Table 4:** The average percent growth inhibition of the triazoles (**4a–j**) and imatinib at 10 µM.

Panels	4a	4b	4c	4d	4e	4f	4g	4h	4i	4j	Imatinib
*Leukemia*	−8.21	−9.90	−6.38	−4.75	−3.89	−2.86	−3.13	−2.86	6.56	0.9	**9**
*Non-small cancer cell*	4.47	3.49	1.40	6.28	3.44	0.60	0.38	6.64	9.12	5.15	**15.68**
*Colon cancer*	−6.36	−9.55	−9.88	−5.92	−5.76	−6.68	−9.39	−7.58	−8.08	−6.26	**5.34**
*CNS cancer*	1.75	0.92	0.55	4.07	8.13	-1.42	1.52	1.95	**8.19**	3.38	5.8
*Melanoma*	−4.08	−4.25	−3.11	−1.30	−3.43	−2.90	−4.53	−0.46	**3.61**	−1.31	−0.87
*Ovarian cancer*	−6.08	−3.03	−5.96	−1.86	−6.49	−4.64	−8.5	−2.50	**3.66**	−5.09	−7.16
*Renal cancer*	−7.54	−7.2	−6.48	−9.77	−0.49	−5.35	−6.08	−4.39	−1.52	−5.87	**3.25**
*Prostate cancer*	−4.93	−9.62	−1.84	0.83	4.72	−14.08	−2.88	−17.08	−1.74	−7.28	**12.5**
Breast cancer	1.66	0.35	−3.48	7.76	1.078	−7.08	−4.05	−1.05	−0.43	1.57	**12.15**

Bold font represents the best result.

**Table 5:** Molecular docking studies of oxadiazoles against tubulin.

S. No.	Compound	PDB ID: 5LYJ		
		Docking Score	Emodel Score	Interaction
1	**4a**	−7.582	−61.616	H-bond (Asn258, 2.47 Å)
2	**4b**	−7.172	−61.018	H-bond (Asn258, 2.33 Å); halogen bond (Cys241, 3.09 Å)
3	**4c**	−7.688	−62.646	−
4	**4d**	−6.502	−58.514	H-bond (Asn258, 2.36 Å)
5	**4e**	−7.899	−65.212	H-bond (Asn258, 2.34 Å); halogen bond (Val315, 3.47 Å)
6	**4f**	−8.264	−66.047	H-bond (Asn258, 2.72 Å)
7	**4g**	−8.341	−74.031	H-bond (Asn258, 2.76 Å)
8	**4h**	−7.788	−57.121	−
9	**4i**	−8.149	−64.382	H-bond (Asn258, 2.43 Å)
10	**4j**	−7.912	−61.999	H-bond (Asn258, 2.38 Å); halogen bond (Val315, 3.47 Å)

**Table 6:** ADME and toxicity prediction studies of triazoles (**4a–j**).

S. No.	Compound	% ABS	Volume	TPSA	NROTB	HBA (<10)	HBD (<5)	Log *p* (<5)	MW (500)	BBB Permeability	Lipinski’s Violation (≤1)	LD_50_ (mg/kg)	Toxicity Class
1	**4a**	90.49	238.75	53.63	3	2	2	2.15	333.16	Yes	0	500	4
2	**4b**	90.50	247.36	53.6	3	2	2	2.33	349.6	Yes	0	500	4
3	**4c**	90.50	250.38	53.6	3	2	2	2.17	329.19	Yes	0	500	4
4	**4d**	87.32	259.37	62.83	4	3	2	2.2	345.19	Yes	0	440	4
5	**4e**	90.50	247.36	53.6	3	2	2	2.44	349.6	Yes	0	500	4
6	**4f**	90.50	250.38	53.6	3	2	2	2.34	329.19	Yes	0	500	4
7	**4g**	87.32	259.37	62.83	4	3	2	2.37	345.19	Yes	0	440	4
8	**4h**	90.50	266.94	53.6	3	2	2	2.48	343.22	Yes	0	500	4
9	**4i**	90.50	266.94	53.6	3	2	2	2.36	343.22	Yes	0	500	4
10	**4j**	90.50	252.29	53.6	3	2	2	2.36	367.6	Yes	0	500	4

## Data Availability

The Supplementary Materials to this article contain information that supports the findings of this study.

## Supplementary Materials

The following supporting information can be downloaded at https://www.mdpi.com/article/10.3390/molecules28196936/s1: General method for the synthesis of intermediate compounds, **2a–j** and **3a–j**; Figures S1–S15: NMR and mass spectra of some of the compounds; Figures S16–S25: Anticancer data of compound **4a–j** against 58 cancer cell lines at 10 µM.

## Sample Availability

Samples of the compounds are available from the authors.
