# Supplementary material for: Synthesis, Anticancer Activity, and In Silico Studies of 5-(3-Bromophenyl)-N-aryl-4H-1,2,4-triazol-3-amine Analogs
Source: Molecules. 2023 Oct 5;28(19):6936. doi: 10.3390/molecules28196936 (PMC10574406; doi:10.3390/molecules28196936)
Supplement: Supplementary file 1 [file molecules-28-06936-s001.zip › molecules-2602443-supplementary.pdf]

Synthesis, Anticancer Activity and *In-Silico* Studies of 5-(3-bromophenyl)-*N*-aryl-4*H*-1,2,4-triazol-3-amines

Mohamed Jawed Ahsan [1,†], Krishna Gautam [1,†], Amena Ali [2], Abuzer Ali [3], Abdulmalik Saleh Alfawaz Altamimi [4,*], Salahuddin [5], Manal A. Alossaimi [4], S.V.V.N.S.M. Lakshmi [6], Md. Faiyaz Ahsan [7]

[1] Department of Pharmaceutical Chemistry, Maharishi Arvind College of Pharmacy, Ambabari Circle, Jaipur, Rajasthan 302039, India

[2] Department of Pharmaceutical Chemistry, College of Pharmacy, Taif University, P.O. Box 11099, Taif 21944 Saudi Arabia

[3] Department of Pharmacognosy, College of Pharmacy, Taif University, P.O. Box 11099, Taif 21944 Saudi Arabia

[4] Department of Pharmaceutical Chemistry, College of Pharmacy, Prince Sattam Bin Abdulaziz University, Al-Kharj 11942, Saudi Arabia

[5] Department of Pharmaceutical Chemistry, Noida Institute of Engineering and Technology (Pharmacy Institute), Knowledge Park-2, Greater Noida 201306, Uttar Pradesh India

[6] Department of Pharmacognosy, Vishnu Institute of Pharmaceutical Education & Research, Narsapur, Medak Dist., Telangana 502 313, India

[7] Department of Chemistry, Bihar National College, Patna, Bihar 800 004, India

* Correspondence: as.altamimi@psau.edu.sa (A.S.A.A.)

† These authors contributed equally to this work.

SUPPLEMENTARY MATERIALS

Materials and Methods

Chemistry

All the chemicals were of synthetic grades. The melting point were recorded by open capillary method and progress of reaction analyzed by thin layer chromatography plate (TLC Silica gel 60 F254). The nuclear magnetic resonance (NMR) and mass spectra were recorded on a Bruker AC 300 MHz spectrometer and waters ACQUITY TQD respectively.

*General method for the synthesis of substituted phenyl urea (**2a-j**)*

An amount substituted aniline (**1a-j**) (0.1 mol) was dissolved in 20 mL glacial acetic acid and make up the volume up to 100 mL with hot water. An equimolar amount of sodium cyanate (0.1 mol; 6.5 g) was dissolved in hot water and the solution was added in solution of substituted aniline with continuous stirring on magnetic stirrer at room temperature. The obtained precipitate was allowed to standby for 30 min and then dipped into the cold-water bath for another 30 min, filtered, washed, filter, dried, and re-crystallized from hot water to obtain substituted phenyl urea (**2a-j**) [25].

*General method for the synthesis of N-substituted phenyl hydrazinecarboxamide (**3a-j**)*

An equimolar amount of substituted phenyl urea (**2a-j**) (7.5 mmol) and hydrazine hydrate (7.5 mmol; ~3.75 mL) was dissolved in 50 mL absolute ethanol and refluxed for 24 h. The reaction mixture was then concentrated, cooled, poured into the crushed ice, filtered, washed with water, dried, and re-crystallized with absolute ethanol to obtain *N*-substituted phenyl hydrazinecarboxamide (**3a-j**) [25].

Developmental Therapeutics Program One Dose Mean Graph	NSC: D-844100 / 1	Conc: 1.00E-5 Molar	Test Date: Apr 10, 2023
	Experiment ID: 2304OS17		Report Date: May 05, 2023

Panel/Cell Line	Growth Percent
**Leukemia**	
CCRF-CEM	108.27
HL-60(TB)	113.71
K-562	112.26
MOLT-4	107.03
RPMI-8226	105.59
SR	102.38
**Non-Small Cell Lung Cancer**	
A549/ATCC	103.16
EKVX	78.28
HOP-62	101.06
NCI-H226	93.31
NCI-H23	88.70
NCI-H322M	94.81
NCI-H460	107.43
NCI-H522	97.48
**Colon Cancer**	
COLO 205	110.19
HCC-2998	115.61
HCT-116	97.94
HCT-15	104.59
HT29	105.50
KM12	105.22
SW-620	105.46
**CNS Cancer**	
SF-268	106.54
SF-295	95.30
SF-539	100.77
SNB-19	101.28
SNB-75	85.12
U251	100.51
**Melanoma**	
LOX IMVI	102.66
MALME-3M	98.54
M14	105.99
MDA-MB-435	106.28
SK-MEL-2	113.88
SK-MEL-28	113.30
SK-MEL-5	99.60
UACC-257	109.84
UACC-62	86.63
**Ovarian Cancer**	
IGROV1	97.79
OVCAR-3	100.18
OVCAR-4	111.22
OVCAR-5	97.87
OVCAR-8	106.58
NCI/ADR-RES	109.03
SK-OV-3	119.91
**Renal Cancer**	
786-0	113.02
A498	135.10
ACHN	114.05
CAKI-1	85.93
RXF 393	116.32
SN12C	102.39
TK-10	120.17
UO-31	73.32
**Prostate Cancer**	
DU-145	104.93
**Breast Cancer**	
MCF7	87.72
MDA-MB-231/ATCC	90.56
HS 578T	99.43
BT-549	95.99
T-47D	110.59
MDA-MB-468	105.73
Mean	103.14
Delta	29.82
Range	61.78

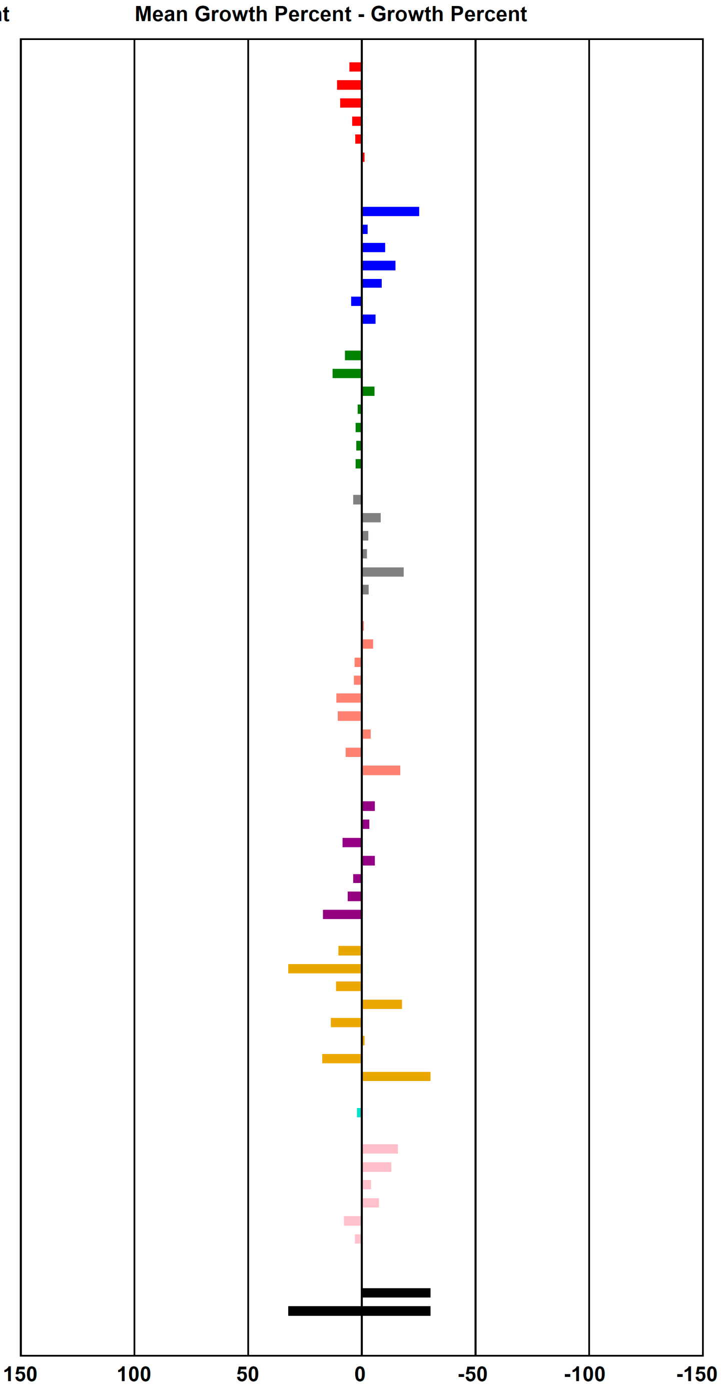

Figure S1. Anticancer data of compound **4a** against 58 cancer cell lines at 10 μM.

Developmental Therapeutics Program One Dose Mean Graph	NSC: D-844101 / 1	Conc: 1.00E-5 Molar	Test Date: Apr 10, 2023
	Experiment ID: 2304OS17		Report Date: May 05, 2023

Panel/Cell Line	Growth Percent
**Leukemia**	
CCRF-CEM	119.99
HL-60(TB)	114.64
K-562	109.82
MOLT-4	108.06
RPMI-8226	105.54
SR	101.37
**Non-Small Cell Lung Cancer**	
A549/ATCC	103.69
EKVX	80.17
HOP-62	95.97
NCI-H226	97.92
NCI-H23	92.08
NCI-H322M	98.59
NCI-H460	109.66
NCI-H522	94.00
**Colon Cancer**	
COLO 205	112.57
HCC-2998	110.62
HCT-116	111.89
HCT-15	102.35
HT29	112.50
KM12	112.61
SW-620	104.34
**CNS Cancer**	
SF-268	100.84
SF-295	95.66
SF-539	99.90
SNB-19	104.36
SNB-75	94.98
U251	98.72
**Melanoma**	
LOX IMVI	101.40
MALME-3M	100.02
M14	108.76
MDA-MB-435	107.80
SK-MEL-2	107.21
SK-MEL-28	112.88
SK-MEL-5	98.99
UACC-257	114.31
UACC-62	86.68
**Ovarian Cancer**	
IGROV1	93.46
OVCAR-3	100.05
OVCAR-4	110.06
OVCAR-5	94.97
OVCAR-8	103.24
NCI/ADR-RES	107.66
SK-OV-3	111.75
**Renal Cancer**	
786-0	103.97
A498	134.22
ACHN	106.61
CAKI-1	82.74
RXF 393	117.62
SN12C	106.19
TK-10	137.39
UO-31	68.86
**Prostate Cancer**	
DU-145	109.62
**Breast Cancer**	
MCF7	80.84
MDA-MB-231/ATCC	89.44
HS 578T	99.85
BT-549	107.15
T-47D	111.95
MDA-MB-468	108.68
Mean	103.75
Delta	34.89
Range	68.53

Mean Growth Percent - Growth Percent

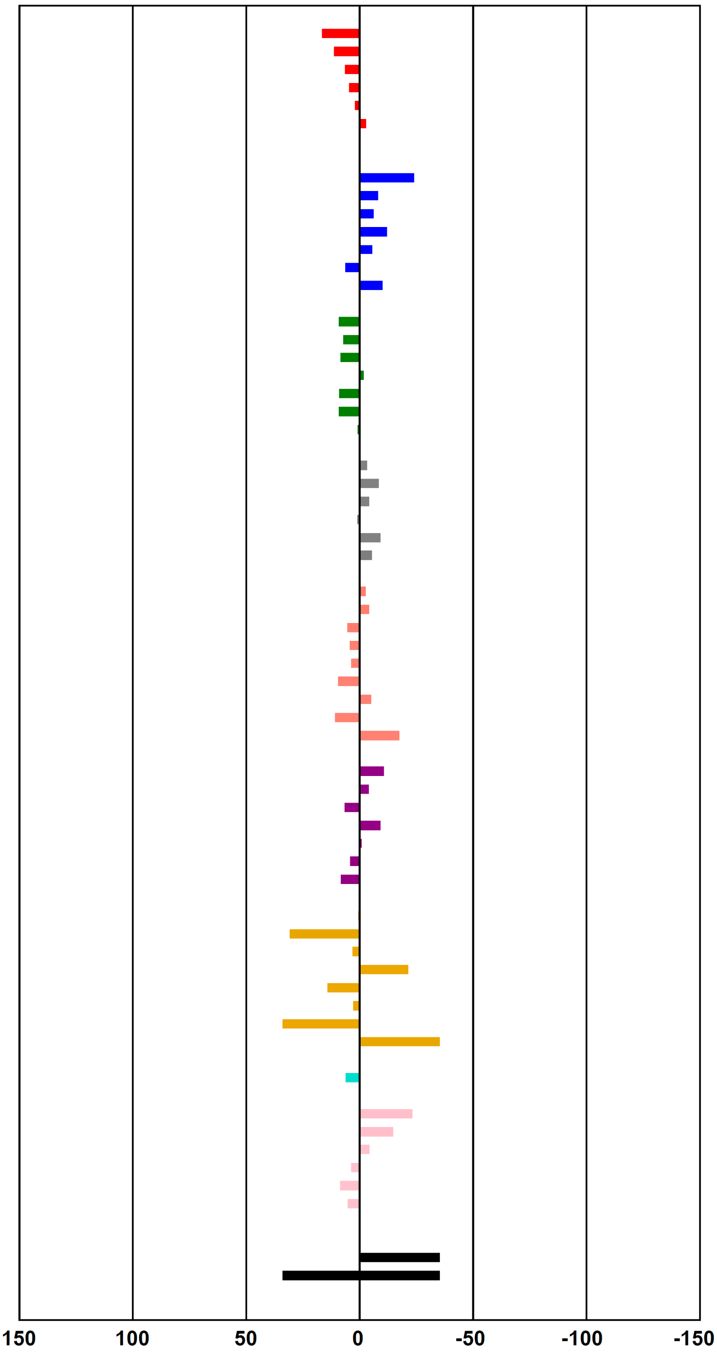

Figure S2. Anticancer data of compound **4b** against 58 cancer cell lines at 10 μM.

Developmental Therapeutics Program
One Dose Mean Graph

NSC: D-844102 / 1	Conc: 1.00E-5 Molar	Test Date: Apr 10, 2023
Experiment ID: 2304OS17		Report Date: May 05, 2023

Panel/Cell Line	Growth Percent
Leukemia	
CCRF-CEM	108.68
HL-60(TB)	111.07
K-562	107.94
MOLT-4	111.15
RPMI-8226	100.45
SR	98.98
Non-Small Cell Lung Cancer	
A549/ATCC	105.55
EKVX	82.04
HOP-62	101.27
NCI-H226	100.07
NCI-H23	93.16
NCI-H322M	104.27
NCI-H460	106.66
NCI-H522	95.75
Colon Cancer	
COLO 205	110.57
HCC-2998	111.63
HCT-116	113.68
HCT-15	107.09
HT29	108.16
KM12	105.97
SW-620	112.08
CNS Cancer	
SF-268	97.75
SF-295	95.57
SF-539	103.01
SNB-19	105.52
SNB-75	93.57
U251	101.28
Melanoma	
LOX IMVI	101.31
MALME-3M	96.32
M14	104.25
MDA-MB-435	105.37
SK-MEL-2	103.44
SK-MEL-28	118.51
SK-MEL-5	99.50
UACC-257	110.07
UACC-62	89.20
Ovarian Cancer	
IGROV1	101.73
OVCAR-3	102.02
OVCAR-4	107.14
OVCAR-5	107.49
OVCAR-8	106.89
NCI/ADR-RES	105.99
SK-OV-3	110.49
Renal Cancer	
786-0	103.28
A498	128.18
ACHN	110.86
CAKI-1	87.09
RXF 393	111.11
SN12C	107.40
TK-10	130.44
UO-31	73.53
Prostate Cancer	
DU-145	101.84
Breast Cancer	
MCF7	81.89
MDA-MB-231/ATCC	94.04
HS 578T	108.56
BT-549	119.87
T-47D	106.74
MDA-MB-468	109.79
Mean	104.09
Delta	30.56
Range	56.91

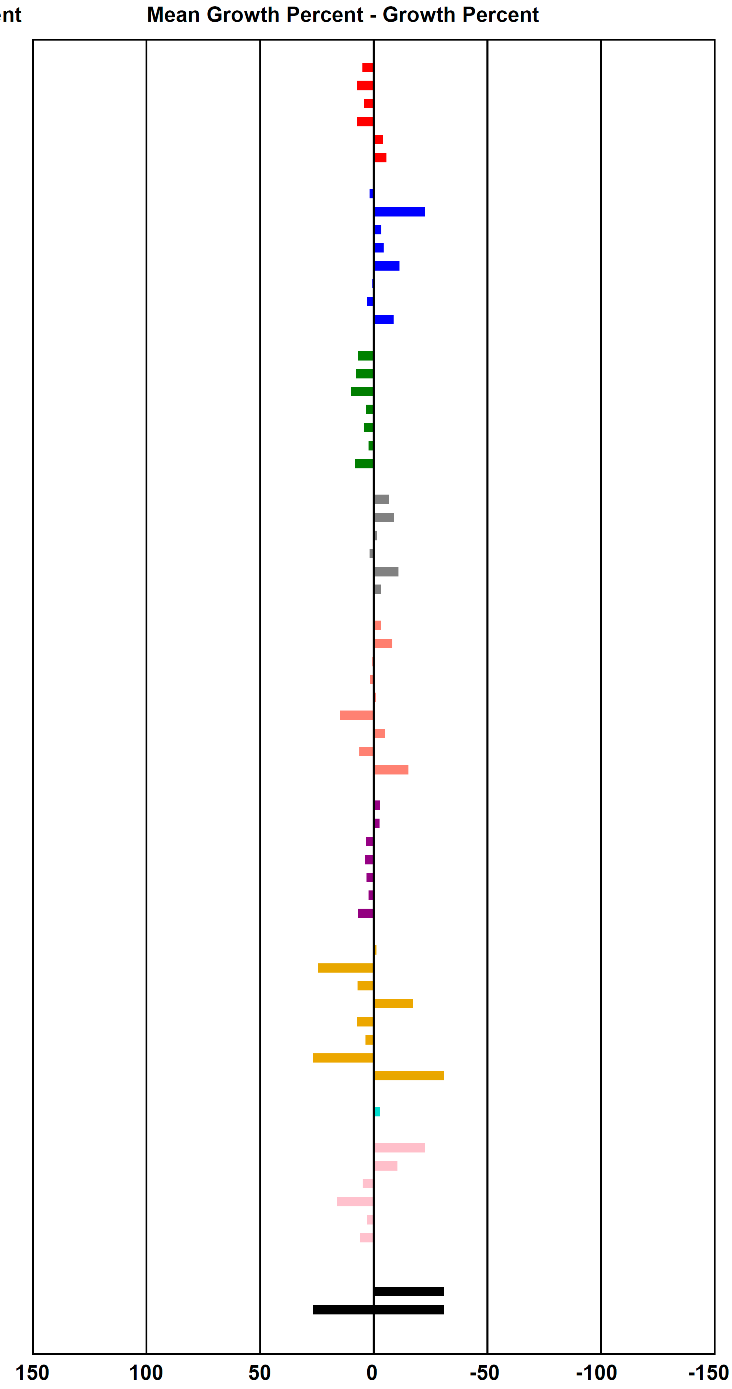

Figure S3. Anticancer data of compound **4c** against 58 cancer cell lines at 10 μM.

Developmental Therapeutics Program<br>One Dose Mean Graph	NSC: D-844103 / 1	Conc: 1.00E-5 Molar	Test Date: Apr 10, 2023
	Experiment ID: 2304OS17		Report Date: May 05, 2023

Panel/Cell Line	Growth Percent
**Leukemia**	
CCRF-CEM	102.09
HL-60(TB)	112.68
K-562	103.69
MOLT-4	110.86
RPMI-8226	98.15
SR	101.01
**Non-Small Cell Lung Cancer**	
A549/ATCC	100.84
EKVX	69.71
HOP-62	92.36
NCI-H226	95.53
NCI-H23	90.01
NCI-H322M	102.66
NCI-H460	102.46
NCI-H522	96.15
**Colon Cancer**	
COLO 205	107.34
HCC-2998	119.85
HCT-116	107.29
HCT-15	101.37
HT29	106.95
KM12	102.43
SW-620	96.23
**CNS Cancer**	
SF-268	95.50
SF-295	96.26
SF-539	103.10
SNB-19	102.63
SNB-75	74.12
U251	103.97
**Melanoma**	
LOX IMVI	100.89
MALME-3M	94.93
M14	112.38
MDA-MB-435	104.17
SK-MEL-2	101.76
SK-MEL-28	108.94
SK-MEL-5	99.12
UACC-257	103.12
UACC-62	86.37
**Ovarian Cancer**	
IGROV1	96.31
OVCAR-3	97.20
OVCAR-4	106.18
OVCAR-5	104.30
OVCAR-8	100.78
NCI/ADR-RES	105.54
SK-OV-3	102.74
**Renal Cancer**	
786-0	108.45
A498	128.70
ACHN	102.05
CAKI-1	82.86
RXF 393	130.25
SN12C	100.67
TK-10	157.41
UO-31	67.80
**Prostate Cancer**	
DU-145	99.17
**Breast Cancer**	
MCF7	64.81
MDA-MB-231/ATCC	88.27
HS 578T	100.87
BT-549	101.94
T-47D	92.60
MDA-MB-468	104.93
Mean	100.88
Delta	36.07
Range	92.60

Mean Growth Percent - Growth Percent

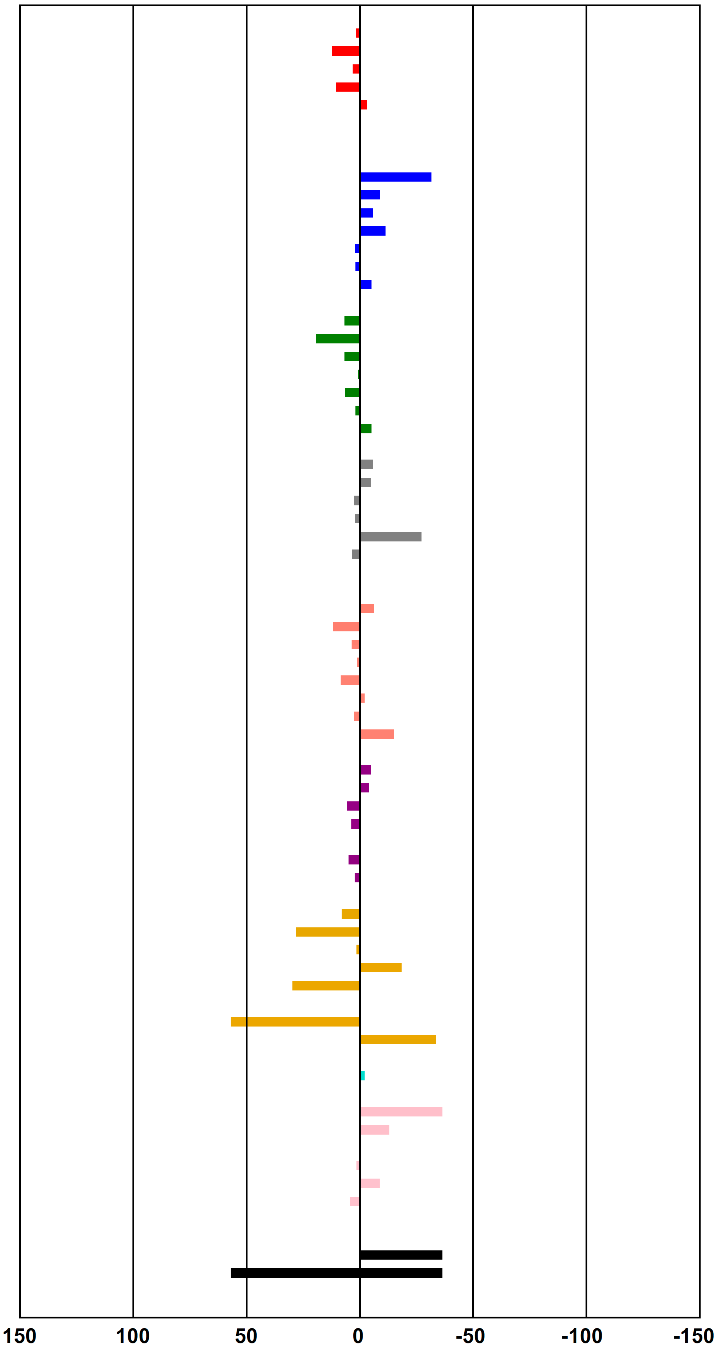

Figure S4. Anticancer data of compound **4d** against 58 cancer cell lines at 10 μM.

Developmental Therapeutics Program	NSC: D-844104 / 1	Conc: 1.00E-5 Molar	Test Date: Apr 10, 2023
One Dose Mean Graph	Experiment ID: 2304OS17		Report Date: May 05, 2023

Panel/Cell Line	Growth Percent
Leukemia	
CCRF-CEM	94.53
HL-60(TB)	110.98
K-562	105.66
MOLT-4	113.17
RPMI-8226	98.41
SR	100.58
Non-Small Cell Lung Cancer	
A549/ATCC	96.10
EKVX	79.19
HOP-62	100.44
NCI-H226	99.37
NCI-H23	93.77
NCI-H322M	103.52
NCI-H460	105.58
NCI-H522	94.51
Colon Cancer	
COLO 205	110.79
HCC-2998	103.10
HCT-116	109.62
HCT-15	102.90
HT29	108.98
KM12	100.98
SW-620	103.98
CNS Cancer	
SF-268	92.22
SF-295	96.71
SF-539	104.07
SNB-19	103.71
SNB-75	58.75
U251	95.73
Melanoma	
LOX IMVI	102.44
MALME-3M	92.71
M14	123.95
MDA-MB-435	104.41
SK-MEL-2	103.40
SK-MEL-28	120.72
SK-MEL-5	99.90
UACC-257	92.92
UACC-62	90.47
Ovarian Cancer	
IGROV1	104.69
OVCAR-3	101.46
OVCAR-4	101.66
OVCAR-5	114.17
OVCAR-8	96.44
NCI/ADR-RES	109.73
SK-OV-3	117.29
Renal Cancer	
786-0	102.12
A498	111.35
ACHN	101.53
CAKI-1	88.17
RXF 393	117.52
SN12C	98.20
TK-10	113.50
UO-31	71.53
Prostate Cancer	
DU-145	95.28
Breast Cancer	
MCF7	88.98
MDA-MB-231/ATCC	95.95
HS 578T	95.00
BT-549	108.77
T-47D	99.07
MDA-MB-468	105.76
Mean	100.97
Delta	42.22
Range	65.20

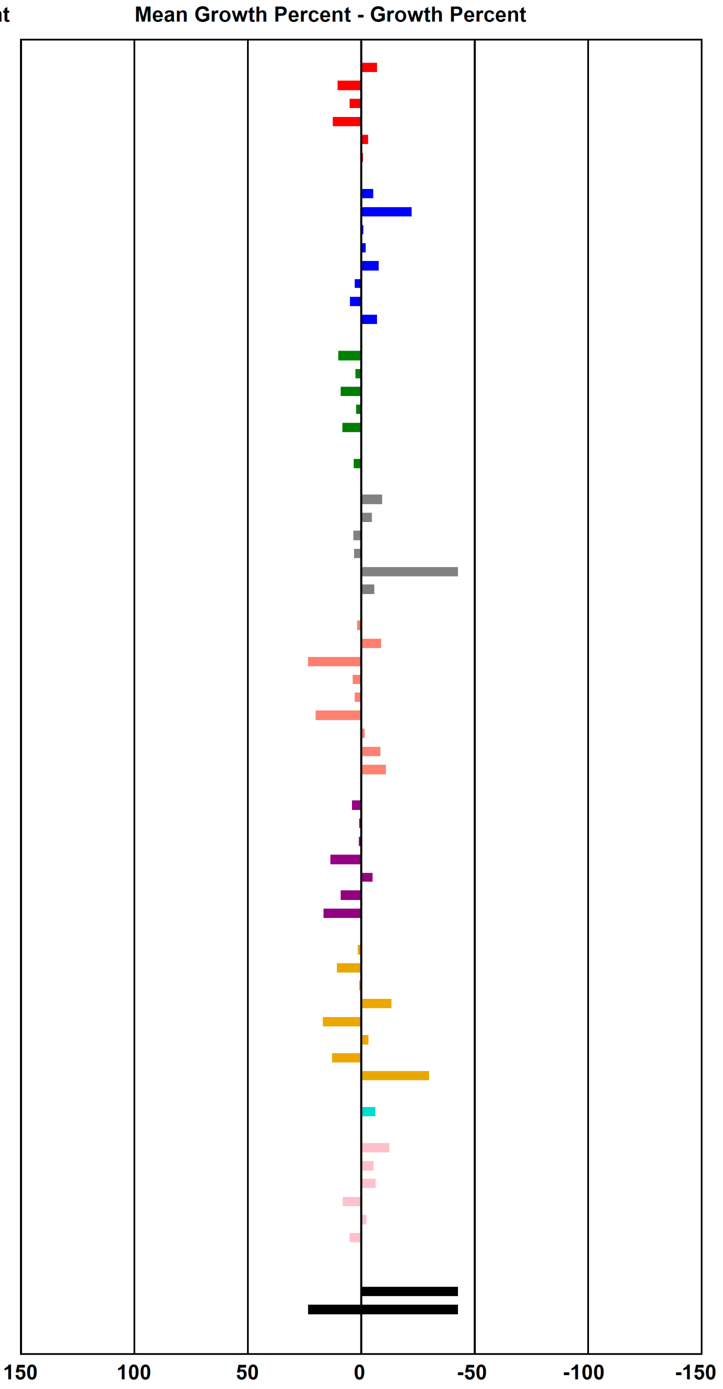

Figure S5. Anticancer data of compound **4e** against 58 cancer cell lines at 10 μM.

Developmental Therapeutics Program<br>One Dose Mean Graph	**NSC:** D-844109 / 1	**Conc:** 1.00E-5 Molar	**Test Date:** Apr 10, 2023
	**Experiment ID:** 2304OS17		**Report Date:** May 05, 2023

Panel/Cell Line	Growth Percent
Leukemia	
CCRF-CEM	100.88
HL-60(TB)	110.97
K-562	102.97
MOLT-4	101.46
RPMI-8226	101.25
SR	99.66
Non-Small Cell Lung Cancer	
A549/ATCC	100.93
EKVX	82.97
HOP-62	95.20
NCI-H226	104.72
NCI-H23	92.05
NCI-H322M	104.54
NCI-H460	111.91
NCI-H522	102.90
Colon Cancer	
COLO 205	113.84
HCC-2998	108.79
HCT-116	110.01
HCT-15	98.06
HT29	110.40
KM12	101.90
SW-620	103.77
CNS Cancer	
SF-268	106.70
SF-295	96.01
SF-539	102.34
SNB-19	97.30
SNB-75	105.10
U251	101.07
Melanoma	
LOX IMVI	99.16
MALME-3M	91.35
M14	105.19
MDA-MB-435	110.41
SK-MEL-2	107.78
SK-MEL-28	115.05
SK-MEL-5	101.68
UACC-257	103.83
UACC-62	91.67
Ovarian Cancer	
IGROV1	93.62
OVCAR-3	108.69
OVCAR-4	114.27
OVCAR-5	103.71
OVCAR-8	103.67
NCI/ADR-RES	105.49
SK-OV-3	103.01
Renal Cancer	
786-0	106.40
A498	126.93
ACHN	111.23
CAKI-1	93.49
RXF 393	108.82
SN12C	105.96
TK-10	127.11
UO-31	62.83
Prostate Cancer	
DU-145	114.08
Breast Cancer	
MCF7	88.37
MDA-MB-231/ATCC	91.79
HS 578T	105.79
BT-549	136.12
T-47D	115.23
MDA-MB-468	105.16
Mean	103.89
Delta	41.06
Range	73.29

Mean Growth Percent - Growth Percent

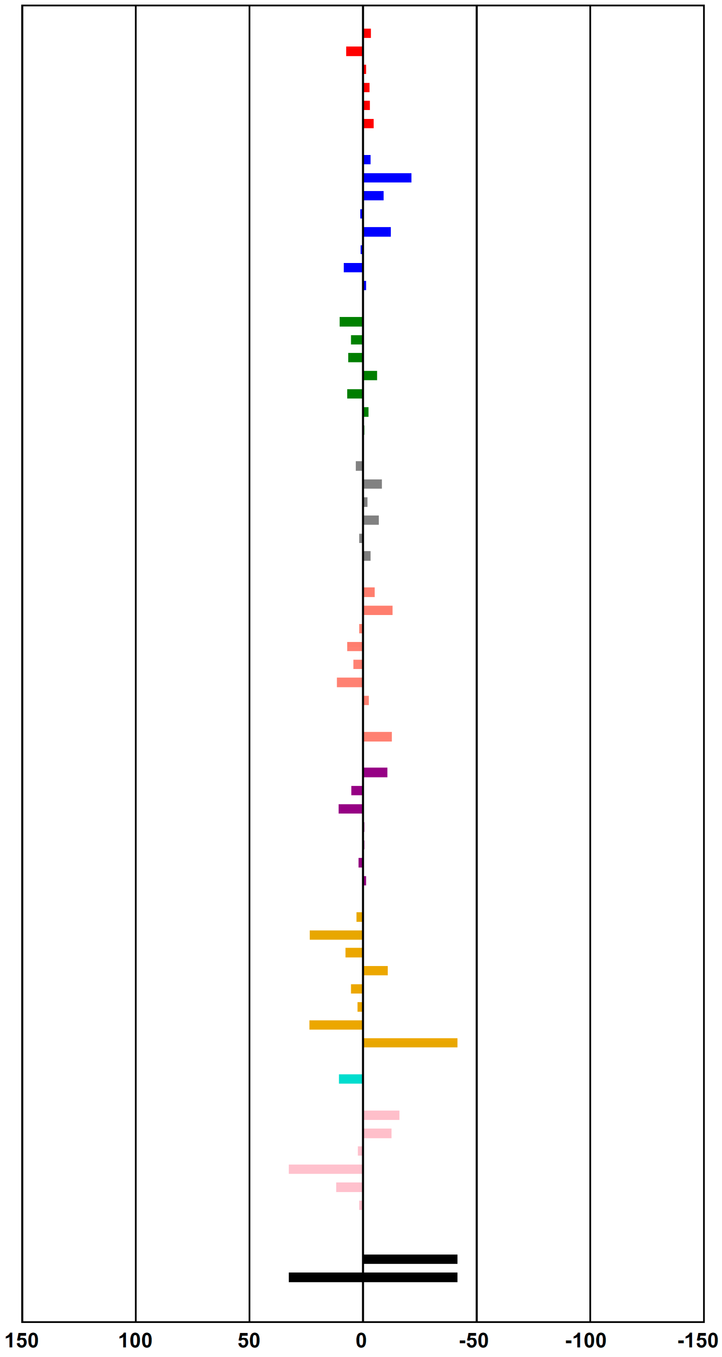

Figure S6. Anticancer data of compound **4f** against 58 cancer cell lines at 10 μM.

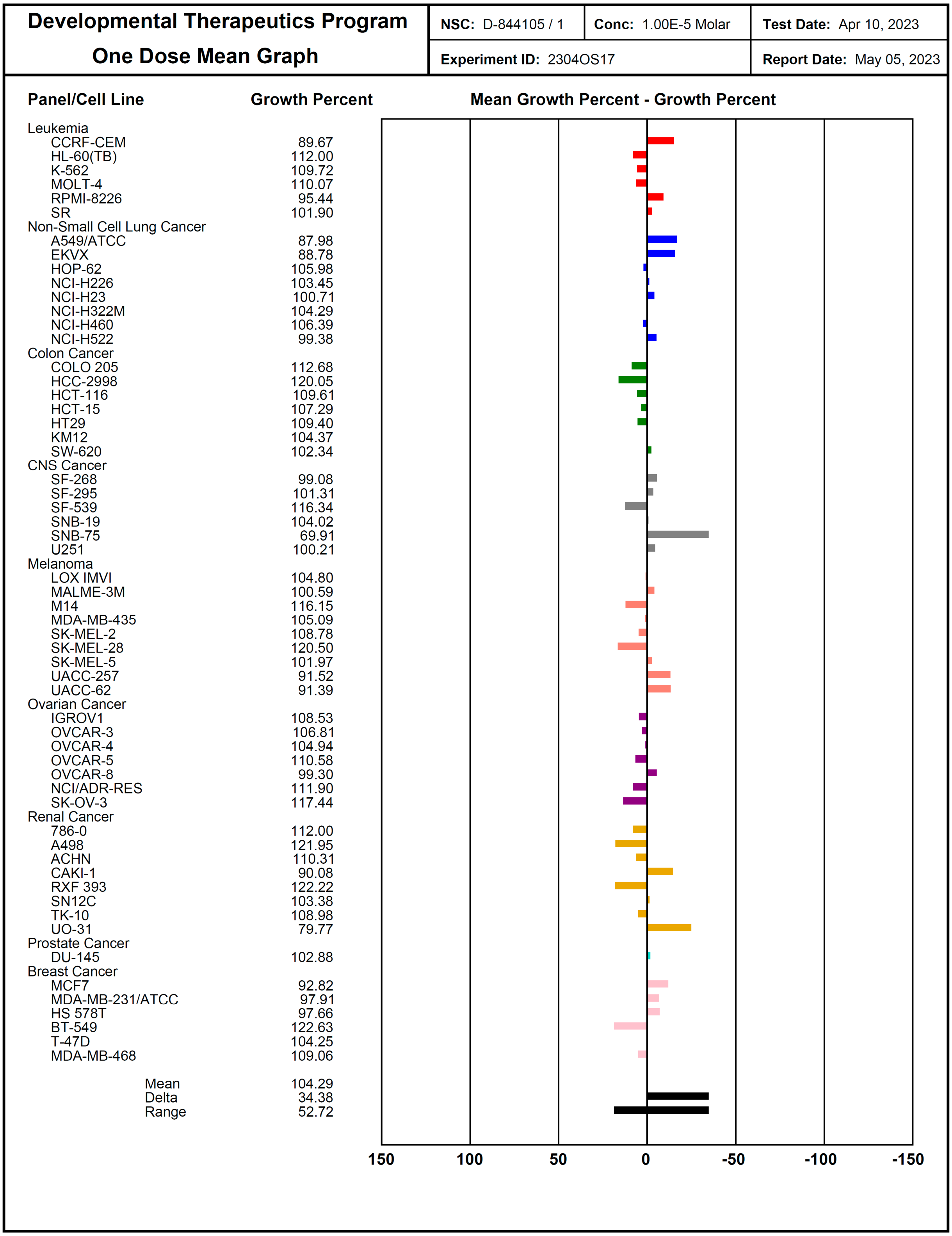

Figure S7. Anticancer data of compound **4g** against 58 cancer cell lines at 10 μM.

Developmental Therapeutics Program One Dose Mean Graph	NSC: D-844108 / 1	Conc: 1.00E-5 Molar	Test Date: Apr 10, 2023
	Experiment ID: 2304OS17		Report Date: May 05, 2023

Panel/Cell Line	Growth Percent
**Leukemia**	
CCRF-CEM	101.76
HL-60(TB)	113.23
K-562	103.20
MOLT-4	102.63
RPMI-8226	99.56
SR	96.81
**Non-Small Cell Lung Cancer**	
A549/ATCC	104.32
EKVX	75.44
HOP-62	88.36
NCI-H226	94.95
NCI-H23	85.13
NCI-H322M	90.57
NCI-H460	108.59
NCI-H522	99.53
**Colon Cancer**	
COLO 205	115.88
HCC-2998	109.12
HCT-116	112.05
HCT-15	101.49
HT29	105.72
KM12	99.84
SW-620	108.99
**CNS Cancer**	
SF-268	106.04
SF-295	91.54
SF-539	95.16
SNB-19	94.59
SNB-75	97.86
U251	103.11
**Melanoma**	
LOX IMVI	92.92
MALME-3M	86.17
M14	105.20
MDA-MB-435	108.55
SK-MEL-2	108.20
SK-MEL-28	112.62
SK-MEL-5	100.20
UACC-257	103.50
UACC-62	86.81
**Ovarian Cancer**	
IGROV1	98.08
OVCAR-3	105.94
OVCAR-4	117.35
OVCAR-5	100.92
OVCAR-8	104.87
NCI/ADR-RES	103.85
SK-OV-3	86.52
**Renal Cancer**	
786-0	100.76
A498	133.39
ACHN	108.67
CAKI-1	94.18
RXF 393	108.85
SN12C	99.06
TK-10	126.78
UO-31	63.43
**Prostate Cancer**	
DU-145	117.08
**Breast Cancer**	
MCF7	88.40
MDA-MB-231/ATCC	88.64
HS 578T	113.29
BT-549	114.36
T-47D	100.03
MDA-MB-468	101.61
Mean	101.48
Delta	38.05
Range	69.96

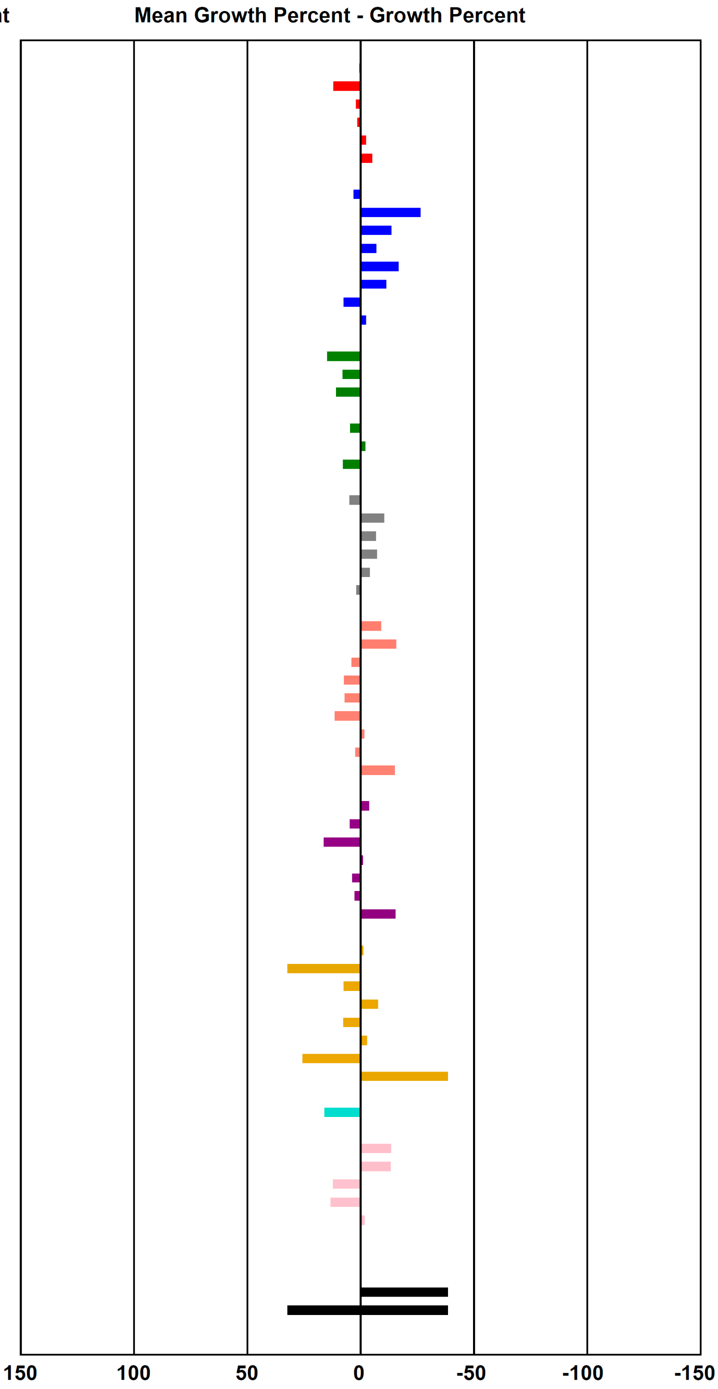

Figure S8. Anticancer data of compound **4h** against 58 cancer cell lines at 10 μM.

Developmental Therapeutics Program One Dose Mean Graph	NSC: D-844106 / 1	Conc: 1.00E-5 Molar	Test Date: Apr 10, 2023
	Experiment ID: 2304OS17		Report Date: May 05, 2023

Panel/Cell Line	Growth Percent
**Leukemia**	
CCRF-CEM	73.08
HL-60(TB)	97.14
K-562	102.19
MOLT-4	97.59
RPMI-8226	95.61
SR	95.05
**Non-Small Cell Lung Cancer**	
A549/ATCC	93.63
EKVX	73.39
HOP-62	84.31
NCI-H226	92.33
NCI-H23	90.72
NCI-H322M	99.97
NCI-H460	100.48
NCI-H522	92.22
**Colon Cancer**	
COLO 205	107.53
HCC-2998	117.60
HCT-116	117.94
HCT-15	105.41
HT29	109.17
KM12	106.75
SW-620	92.19
**CNS Cancer**	
SF-268	95.37
SF-295	97.66
SF-539	101.64
SNB-19	96.04
SNB-75	61.06
U251	99.09
**Melanoma**	
LOX IMVI	95.05
MALME-3M	90.31
M14	98.01
MDA-MB-435	102.27
SK-MEL-2	97.14
SK-MEL-28	112.30
SK-MEL-5	99.65
UACC-257	91.31
UACC-62	81.50
**Ovarian Cancer**	
IGROV1	95.53
OVCAR-3	101.90
OVCAR-4	102.73
OVCAR-5	76.88
OVCAR-8	97.38
NCI/ADR-RES	100.18
SK-OV-3	99.76
**Renal Cancer**	
786-0	101.23
A498	116.19
ACHN	97.50
CAKI-1	82.44
RXF 393	119.36
SN12C	98.18
TK-10	127.43
UO-31	69.86
**Prostate Cancer**	
DU-145	101.74
**Breast Cancer**	
MCF7	91.88
MDA-MB-231/ATCC	82.84
HS 578T	99.85
BT-549	116.88
T-47D	103.99
MDA-MB-468	107.16
Mean	97.48
Delta	36.42
Range	66.37

Mean Growth Percent - Growth Percent

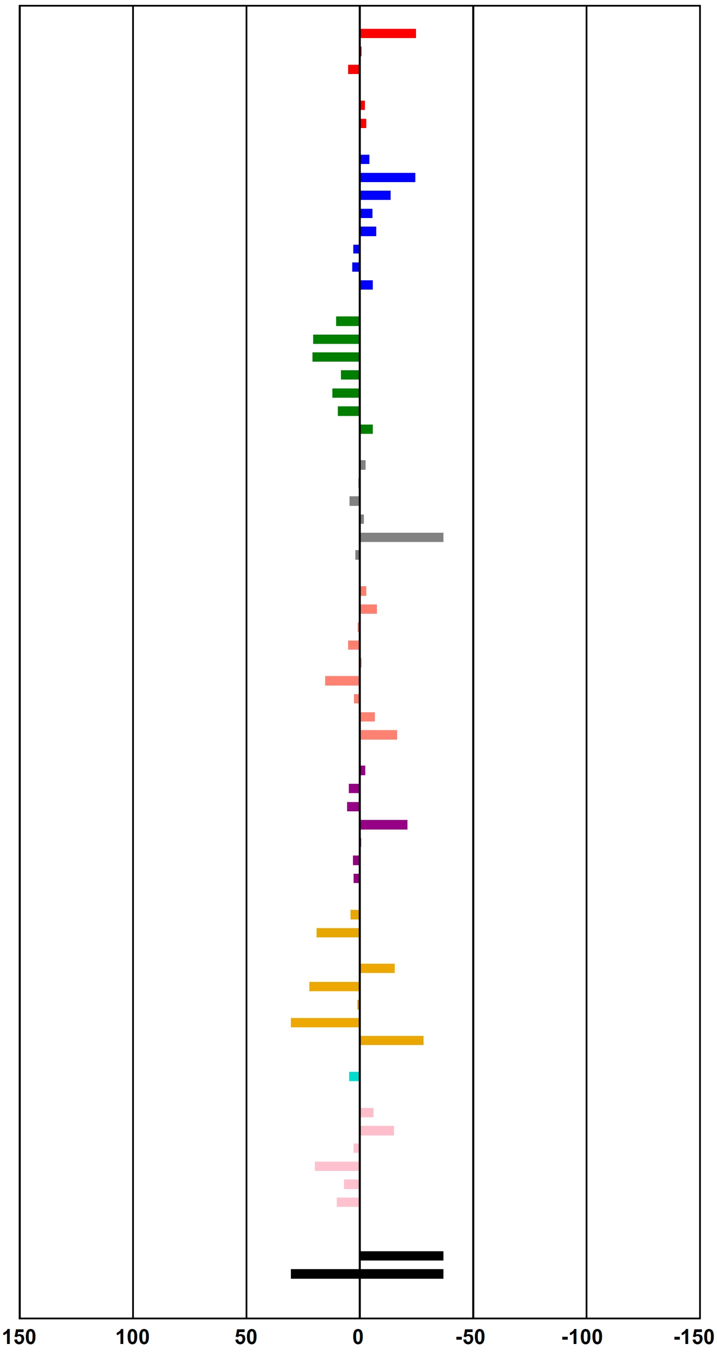

Figure S9. Anticancer data of compound **4i** against 58 cancer cell lines at 10 μM.

Developmental Therapeutics Program One Dose Mean Graph	NSC: D-844107 / 1	Conc: 1.00E-5 Molar	Test Date: Apr 10, 2023
	Experiment ID: 2304OS17		Report Date: May 05, 2023

Panel/Cell Line	Growth Percent
Leukemia	
CCRF-CEM	91.67
HL-60(TB)	114.35
K-562	103.41
MOLT-4	97.05
RPMI-8226	97.90
SR	90.22
Non-Small Cell Lung Cancer	
A549/ATCC	106.05
EKVX	75.43
HOP-62	89.96
NCI-H226	98.69
NCI-H23	86.94
NCI-H322M	99.48
NCI-H460	106.13
NCI-H522	96.13
Colon Cancer	
COLO 205	115.25
HCC-2998	107.20
HCT-116	108.97
HCT-15	98.35
HT29	108.45
KM12	98.24
SW-620	107.34
CNS Cancer	
SF-268	108.16
SF-295	90.80
SF-539	97.27
SNB-19	93.01
SNB-75	87.08
U251	103.42
Melanoma	
LOX IMVI	94.16
MALME-3M	84.86
M14	111.26
MDA-MB-435	108.50
SK-MEL-2	103.77
SK-MEL-28	117.18
SK-MEL-5	100.08
UACC-257	109.37
UACC-62	82.62
Ovarian Cancer	
IGROV1	95.01
OVCAR-3	114.63
OVCAR-4	114.79
OVCAR-5	95.79
OVCAR-8	107.02
NCI/ADR-RES	103.94
SK-OV-3	104.47
Renal Cancer	
786-0	98.23
A498	120.55
ACHN	106.13
CAKI-1	97.73
RXF 393	115.41
SN12C	97.50
TK-10	144.83
UO-31	66.57
Prostate Cancer	
DU-145	107.28
Breast Cancer	
MCF7	75.48
MDA-MB-231/ATCC	88.16
HS 578T	105.98
BT-549	120.26
T-47D	104.12
MDA-MB-468	96.56
Mean	101.19
Delta	34.62
Range	78.26

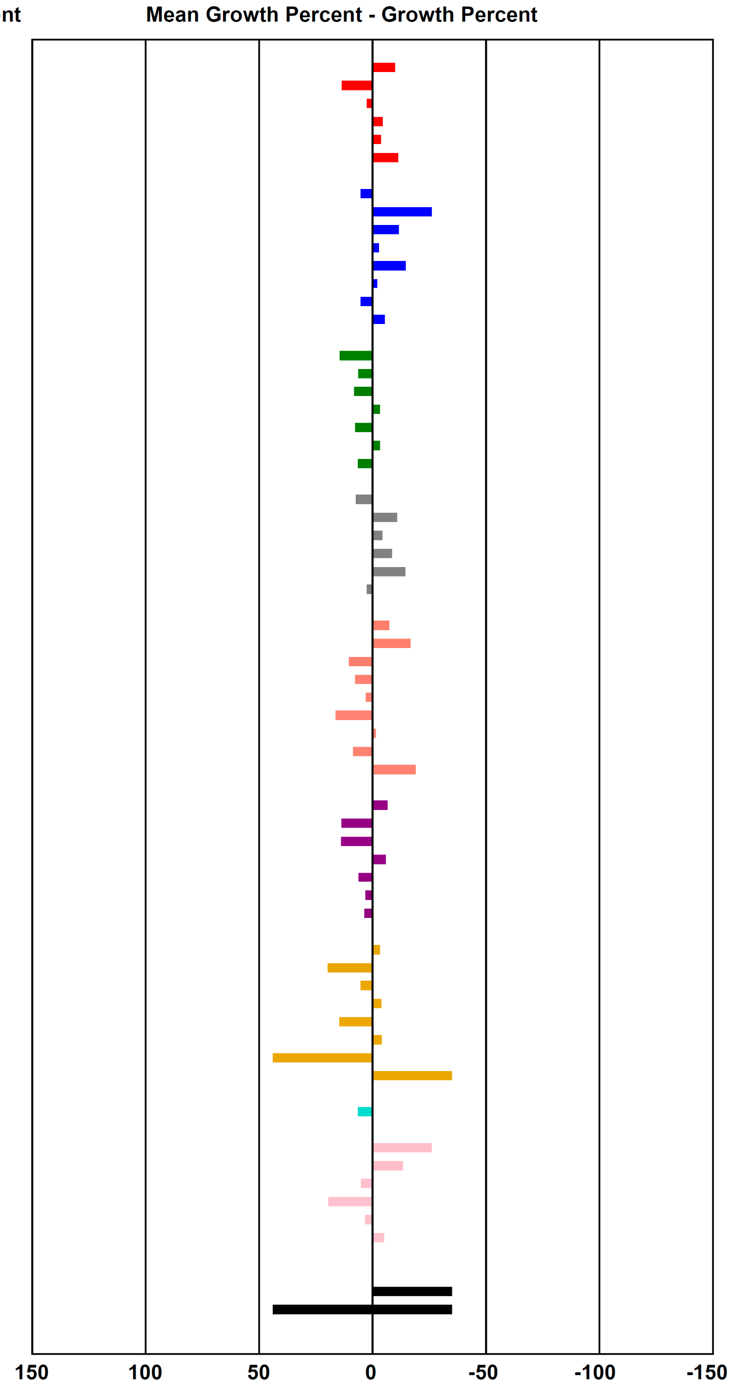

Figure S10. Anticancer data of compound **4j** against 58 cancer cell lines at 10 μM.

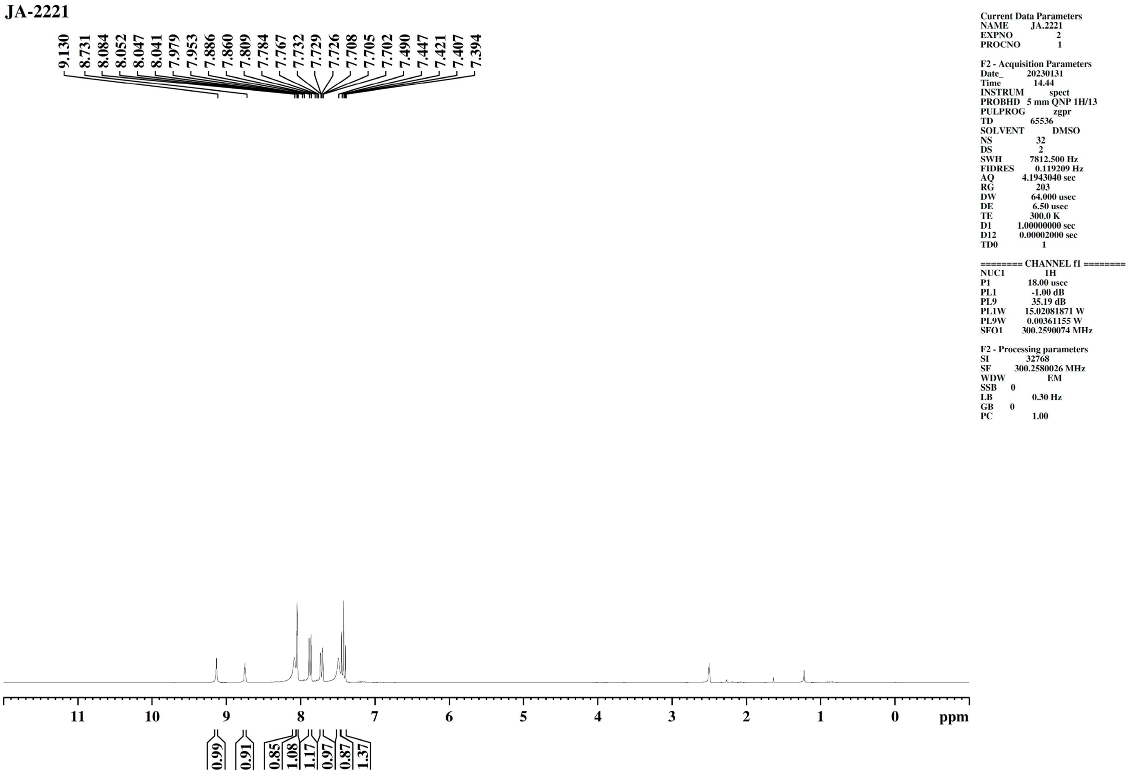

Figure S11. ^{1}H NMR spectra of compound **4a**.

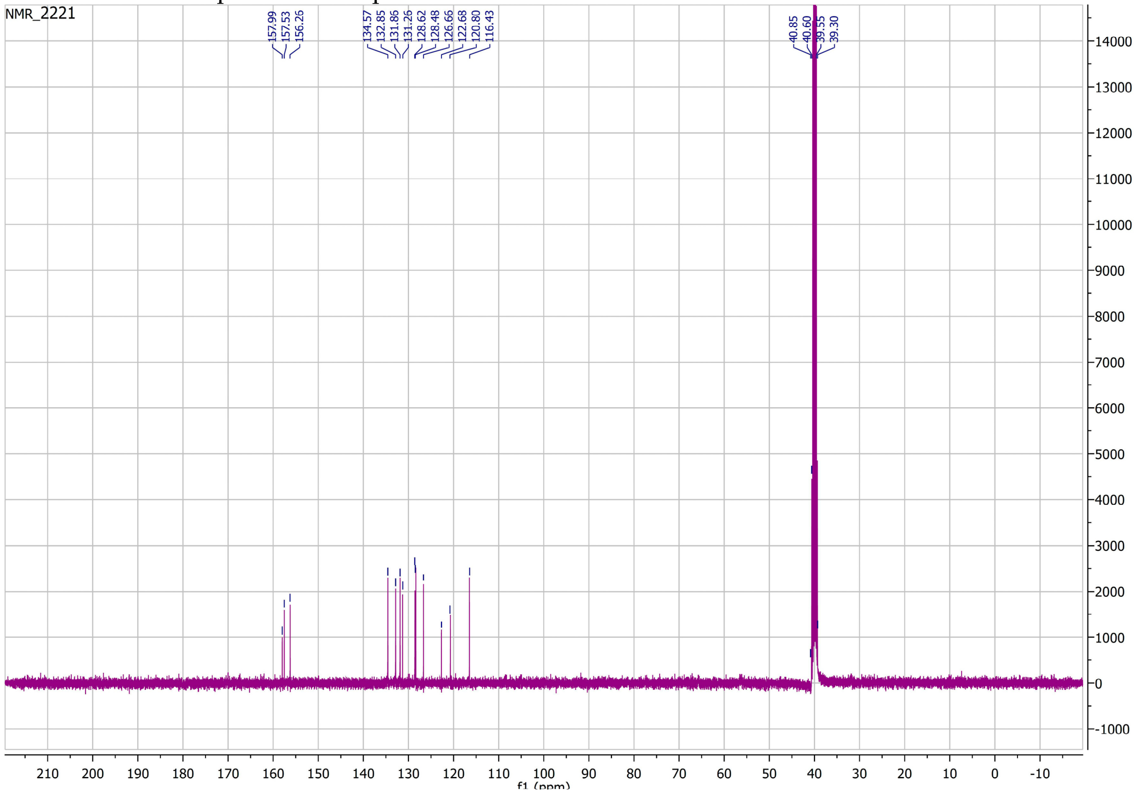

Figure S12. ^{13}C NMR spectra of compound **4a**.

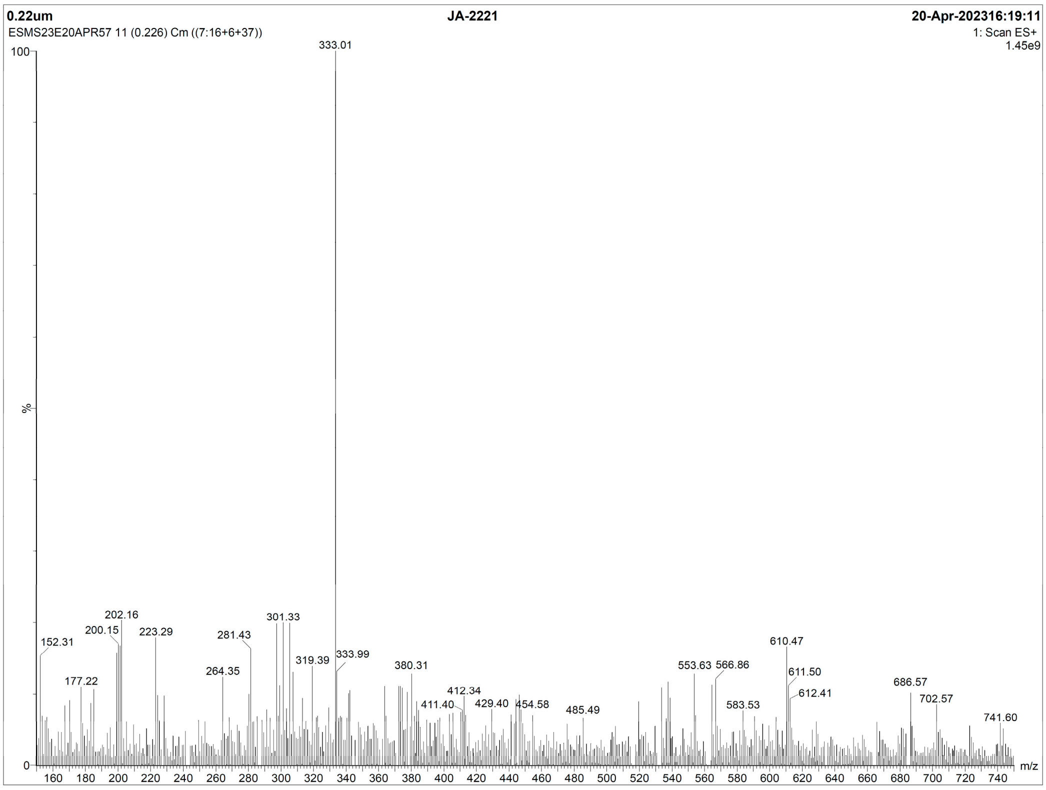

Figure S13. Mass spectra of compound **4a**.

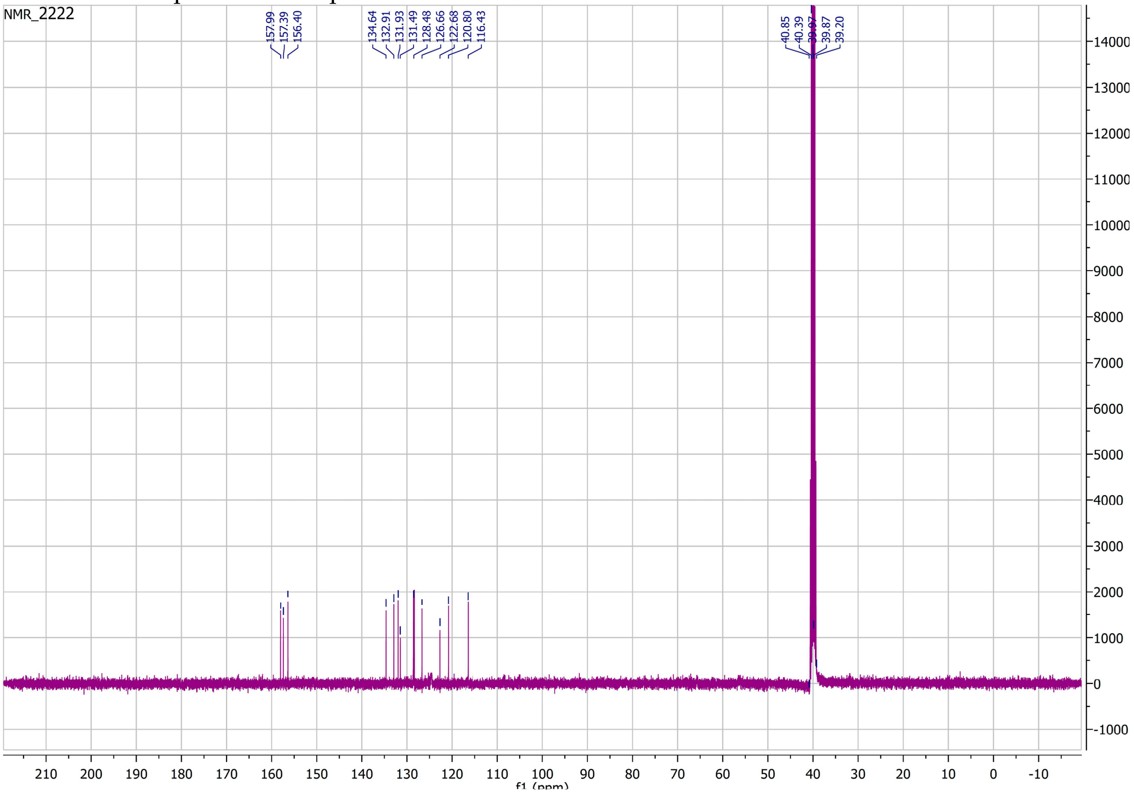

Figure S14. ^{13}C NMR spectra of compound **4b**.

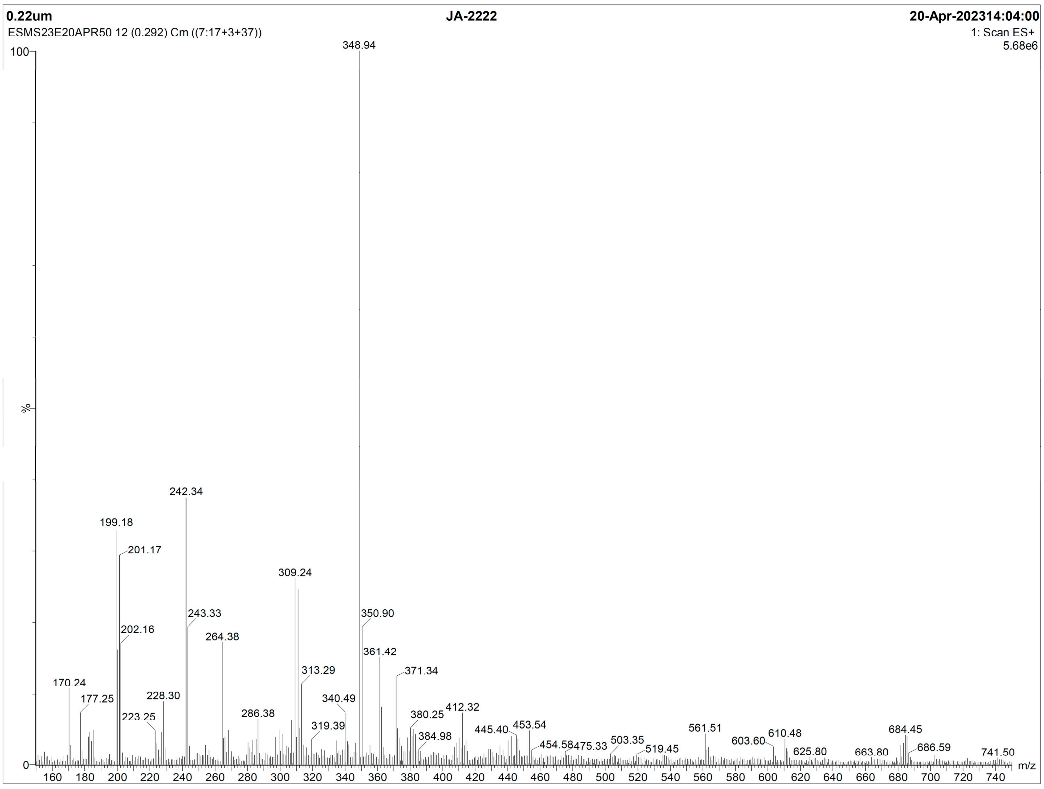

Figure S15. Mass spectra of compound **4b**.

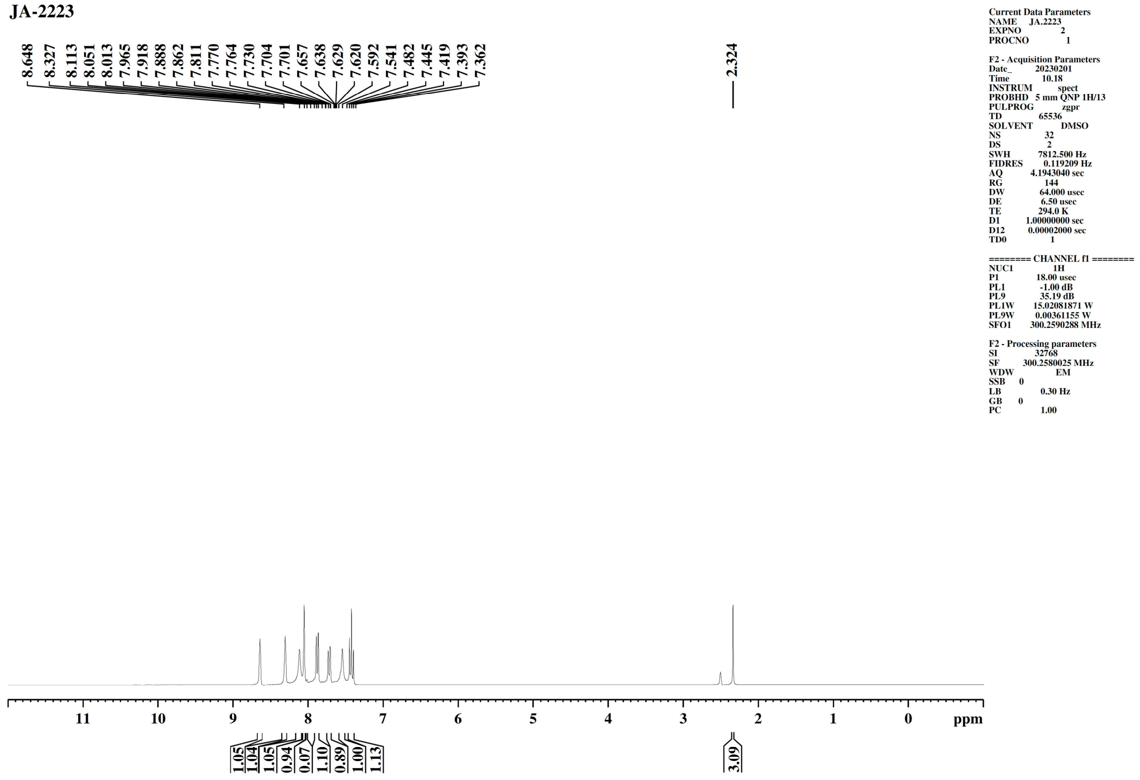

Figure S16. ^{1}H NMR spectra of compound **4c**.

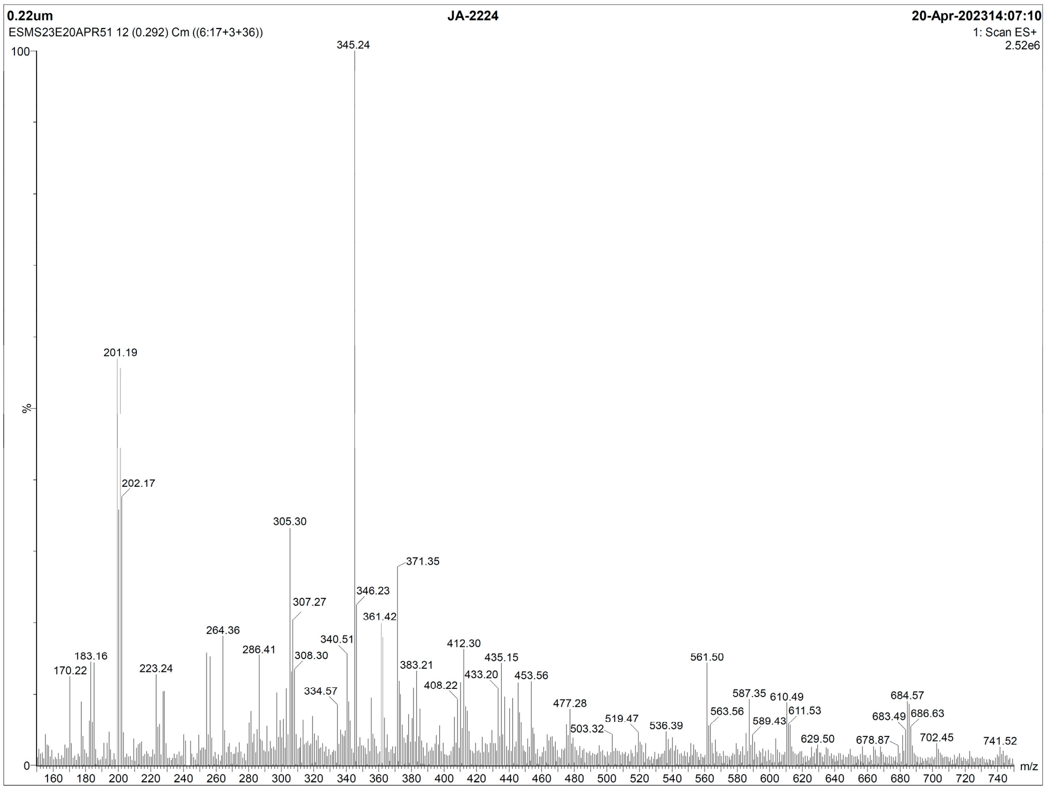

Figure S17. Mass spectra of compound **4d**.

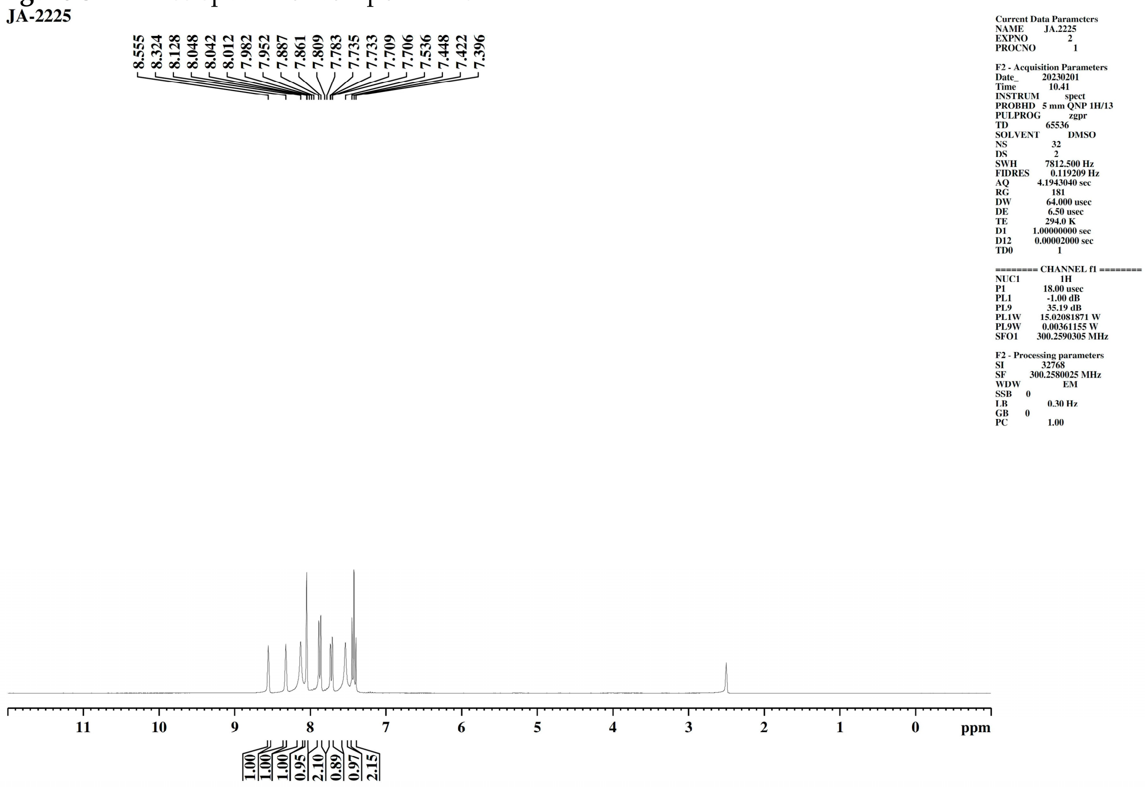

Figure S18. ^{1}H NMR spectra of compound **4e**.

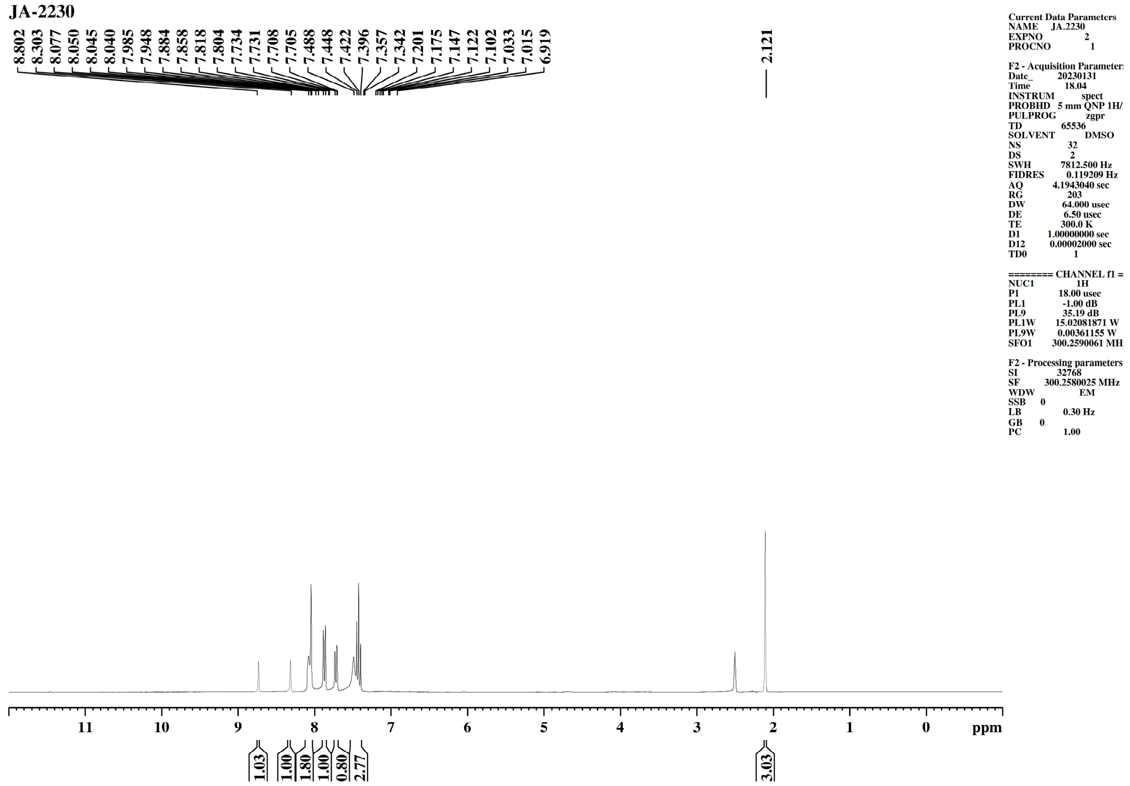

Figure S19. ^{1}H NMR spectra of compound **4f**.

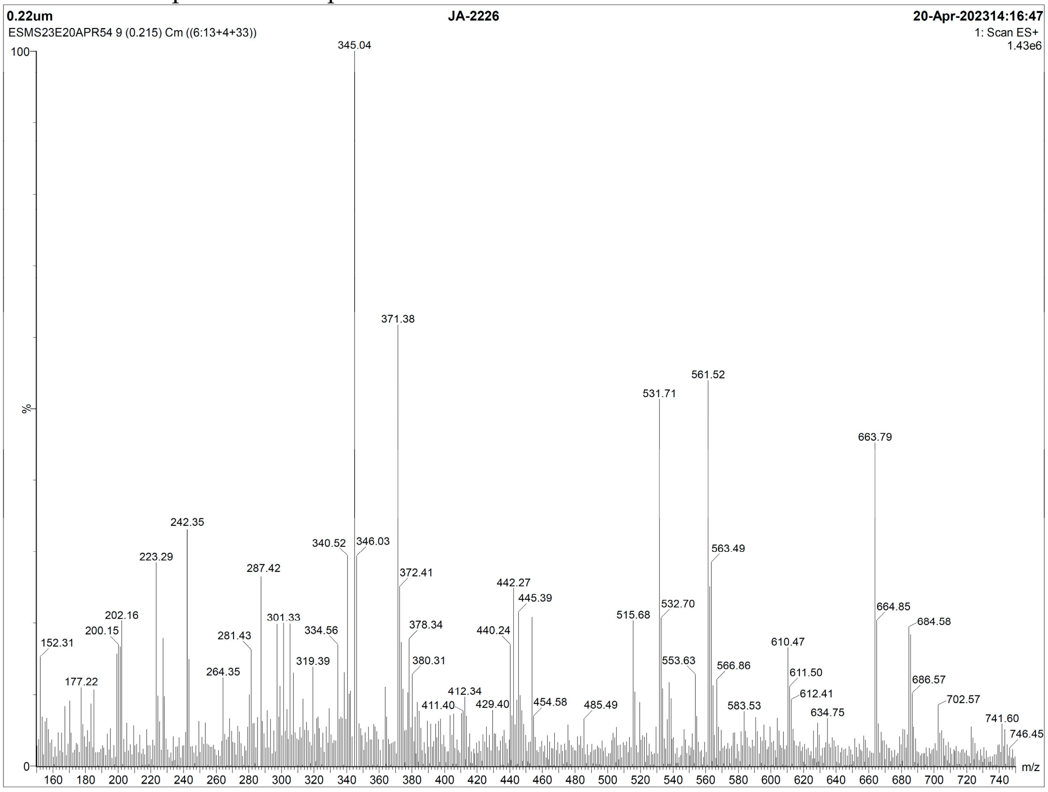

Figure S20. Mass spectra of compound **4g**.

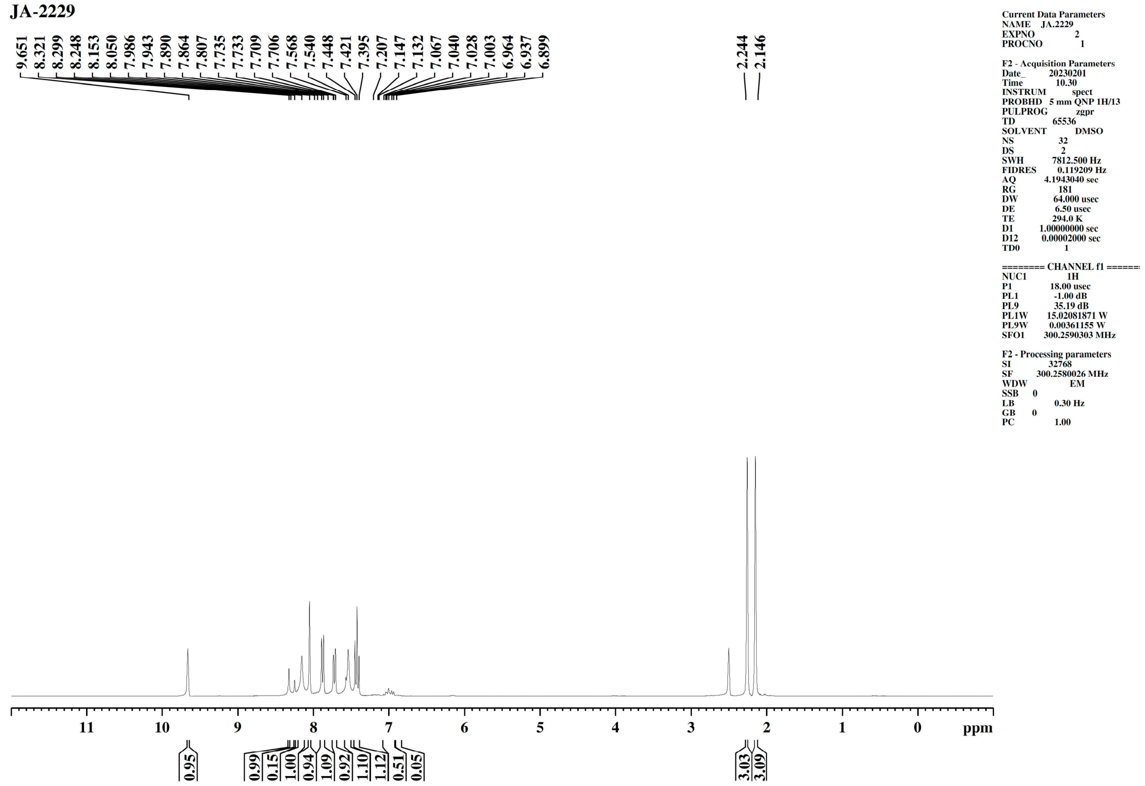

Figure S21. ^{1}H NMR spectra of compound **4h**.

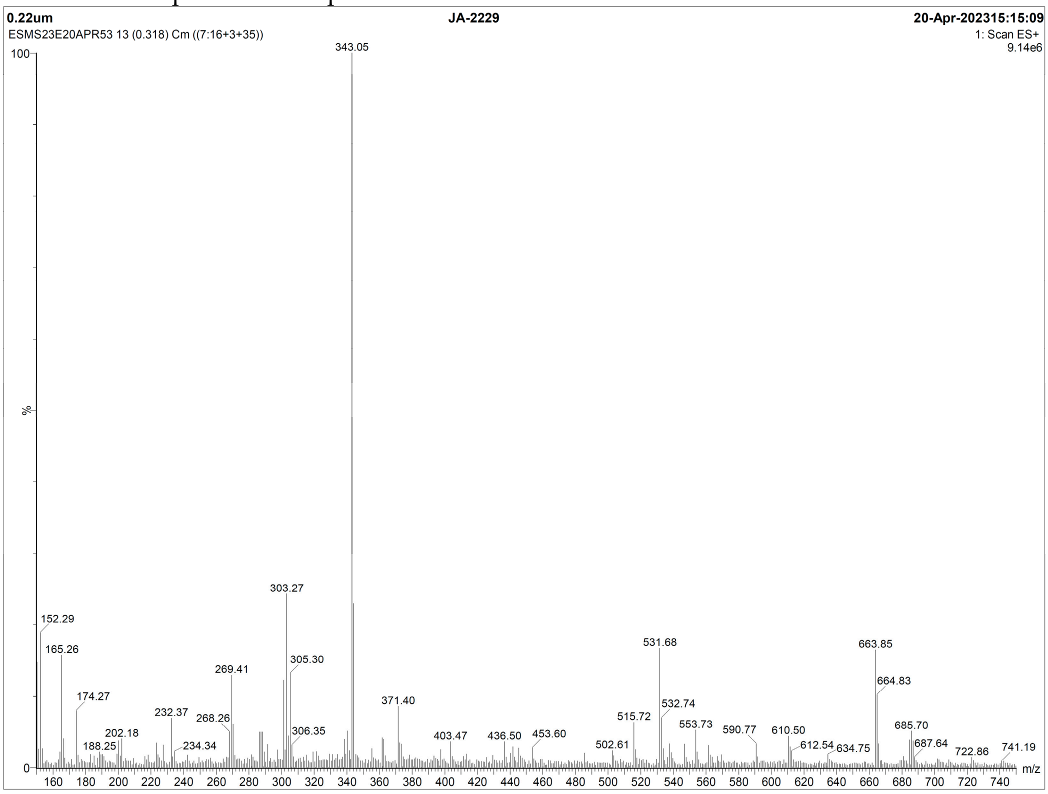

Figure S22. Mass spectra of compound **4h**.

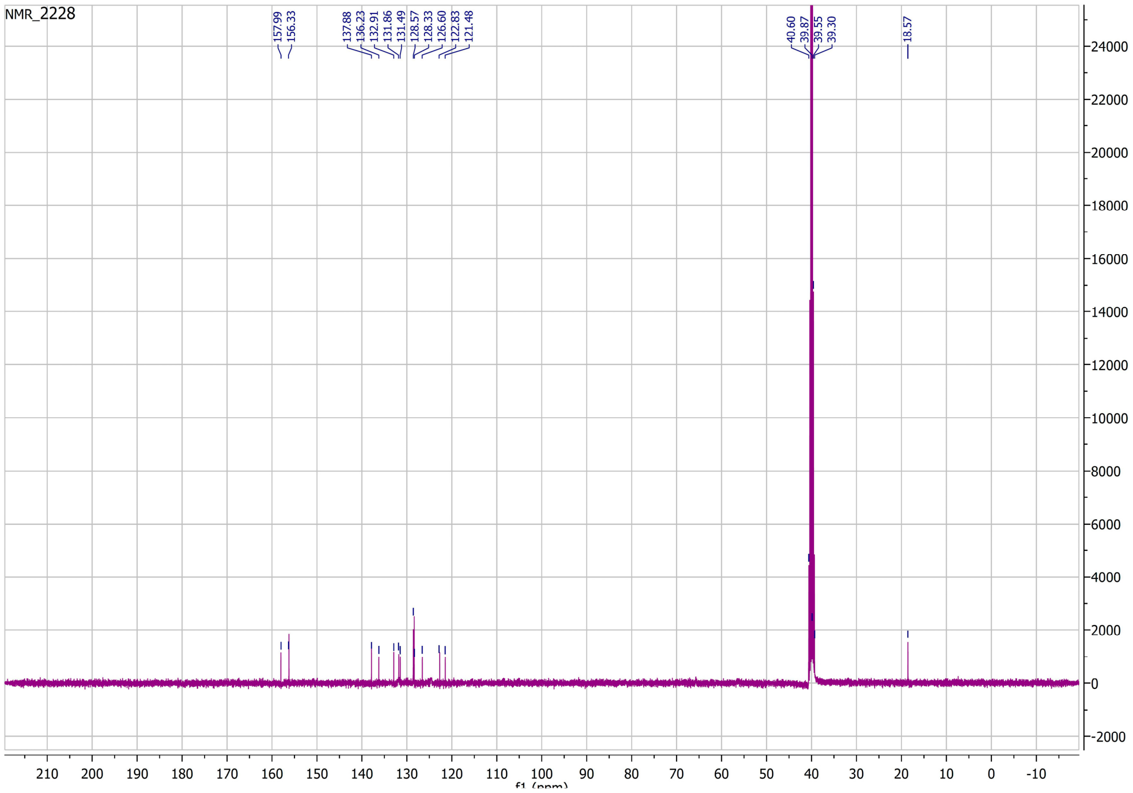

Figure S23. ^{13}C NMR spectra of compound **4i**.

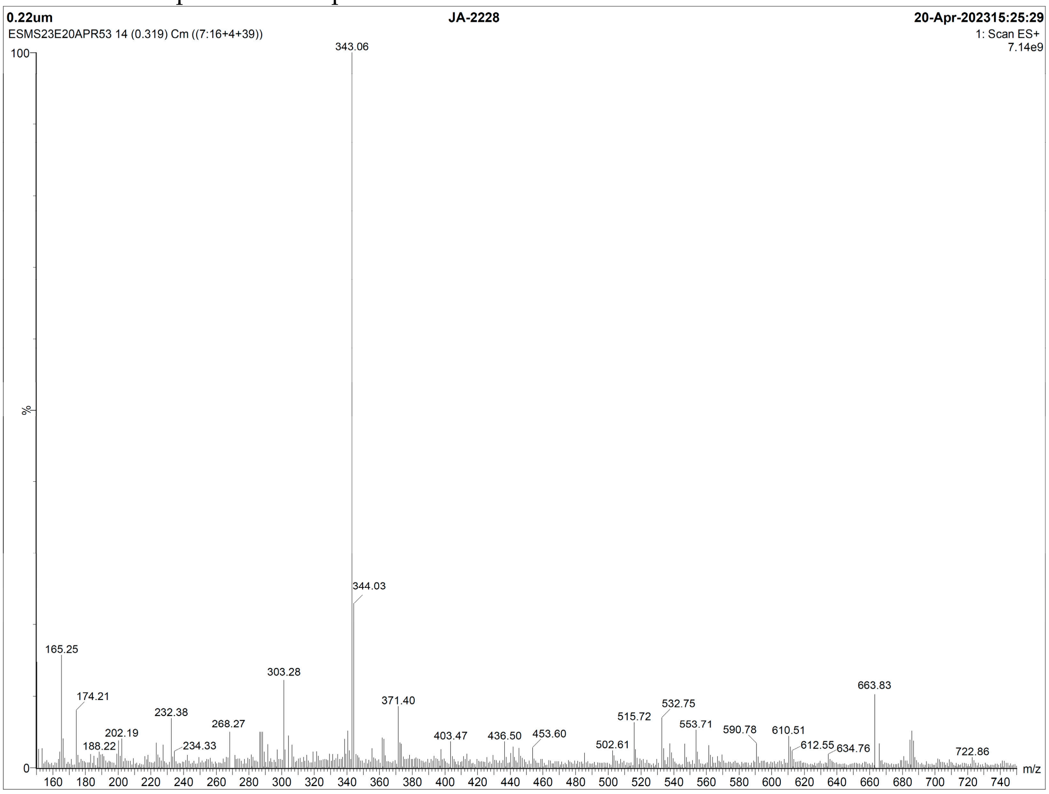

Figure S24. Mass spectra of compound **4i**.

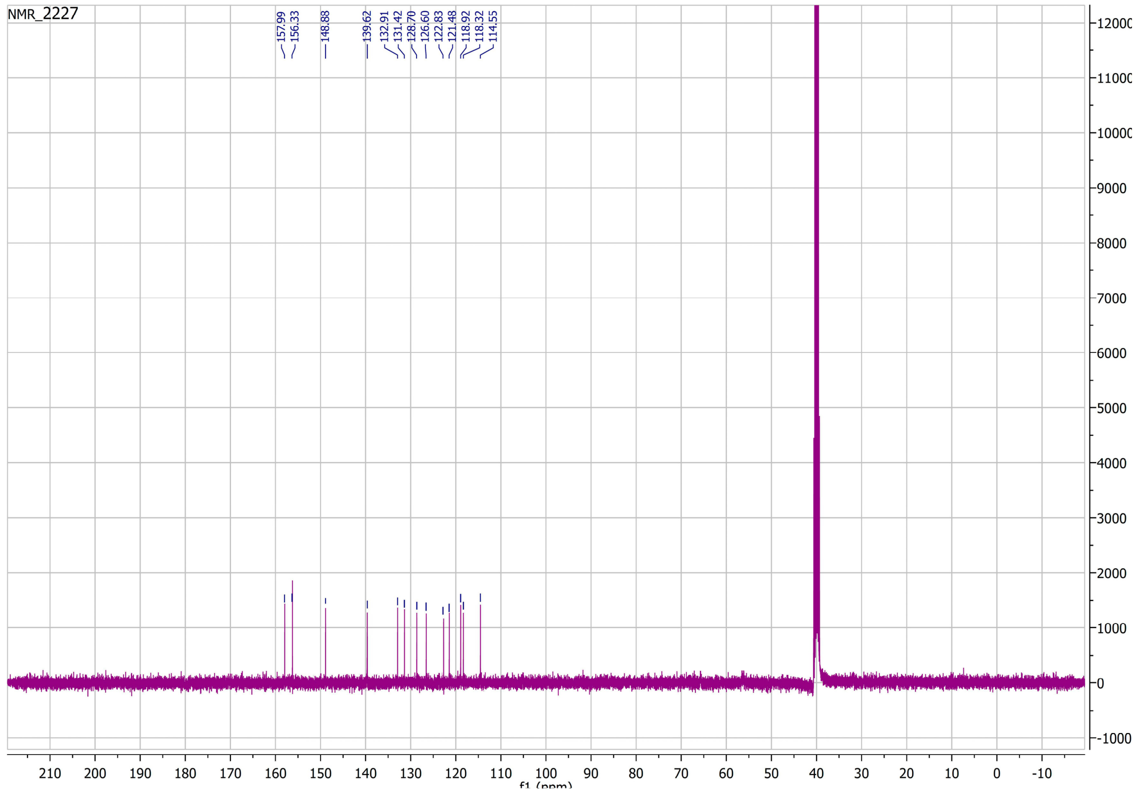

Figure S25. ^{13}C NMR spectra of compound **4j**.
